# Suboptimal human inference can invert the bias-variance trade-off for decisions with asymmetric evidence

**DOI:** 10.1371/journal.pcbi.1010323

**Published:** 2022-07-19

**Authors:** Tahra L. Eissa, Joshua I. Gold, Krešimir Josić, Zachary P. Kilpatrick

**Affiliations:** 1 Department of Applied Mathematics, University of Colorado Boulder, Boulder, Colorado, United States of America; 2 Department of Neuroscience, University of Pennsylvania, Philadelphia, Pennsylvania, United States of America; 3 Department of Mathematics, University of Houston, Houston, Texas, United States of America; 4 Department of Biology and Biochemistry, University of Houston, Houston, Texas, United States of America; 5 Institute of Cognitive Science, University of Colorado Boulder, Boulder, Colorado, United States of America; Durham University, UNITED KINGDOM

## Abstract

Solutions to challenging inference problems are often subject to a fundamental trade-off between: 1) bias (being systematically wrong) that is minimized with complex inference strategies, and 2) variance (being oversensitive to uncertain observations) that is minimized with simple inference strategies. However, this trade-off is based on the assumption that the strategies being considered are optimal for their given complexity and thus has unclear relevance to forms of inference based on suboptimal strategies. We examined inference problems applied to rare, asymmetrically available evidence, which a large population of human subjects solved using a diverse set of strategies that varied in form and complexity. In general, subjects using more complex strategies tended to have lower bias and variance, but with a dependence on the form of strategy that reflected an inversion of the classic bias-variance trade-off: subjects who used more complex, but imperfect, Bayesian-like strategies tended to have lower variance but higher bias because of incorrect tuning to latent task features, whereas subjects who used simpler heuristic strategies tended to have higher variance because they operated more directly on the observed samples but lower, near-normative bias. Our results help define new principles that govern individual differences in behavior that depends on rare-event inference and, more generally, about the information-processing trade-offs that can be sensitive to not just the complexity, but also the optimality, of the inference process.

## Author summary

People use diverse strategies to make inferences about the world around them, often based on limited evidence. Such inference strategies may be simple but prone to systematic errors or more complex and accurate, but such trends need not always be the rule. We modeled and measured how human participants made rare-event decisions in a preregistered, online study. The participants tended to use suboptimal decision strategies that reflected an inversion of the classic bias-variance trade-off: some used complex, nearly normative strategies with mistuned evidence weights that corresponded to relatively high choice biases but lower choice variance, whereas others used simpler heuristic strategies that corresponded to lower biases but higher variance. These relationships illustrate structure in suboptimality that can be used to identify systematic sources of human errors.

## Introduction

Understanding how the brain makes inferences about the world requires first understanding the diversity of strategies individuals use to solve inference problems. One useful approach for understanding this diversity is to assess patterns of errors, which can reflect particular strategies. In general, errors can result from either: 1) bias, which can arise from an incorrect model of the world that produces inferences that are systematically offset from the ground truth; or 2) variability, which can reflect either intrinsic noise or oversensitivity to particular observations (which we refer to as “noise” and “variance,” respectively) and can lead to inferences that are variable over multiple instances of the same problem. Some forms of inference reflect an inherent trade-off between bias and variance[1] that depends on the complexity of the inference process [2, 3]: higher complexity provides more flexibility that tends to decrease bias but incorporates oversensitivity to task-irrelevant variability, whereas lower complexity tends to increase bias but decrease variance. However, this trade-off has typically been considered in the context of inference processes (or “models” in machine learning) that vary in complexity, but are optimized for the given problem and complexity level. Much less understood is whether and how similar trade-offs arise as people solve inference problems using suboptimal strategies [4–6].

To better identify the sources of errors in suboptimal inference, and how these sources of error might relate to the bias-variance trade-off, we examined the choice behavior of human subjects performing a two-alternative forced-choice inference task in which evidence in favor of one alternative was sparse [7]. These inference problems are interesting because they give rise to choice asymmetries; i.e., a tendency to chose one alternative more frequently than the other, even when the alternatives are *a priori* equally likely. We exploited this tendency to identify how subjects’ strategies differed in terms of their resulting choice bias and variance, which were defined with respect to values obtained by the ideal observer performing the (simulated) task under the same conditions. We were particularly interested in how deviations from the ideal observer differed across individual subjects and task conditions, and how these suboptimalities related to the underlying inference strategies that we identified using quantitative model fitting and other methods.

We focused on two classes of strategies whose differences were central to our interpretation of the suboptimal bias-variance trade-off under asymmetric conditions. The first was based on Bayesian principles. We used several related models, each of which produced choice asymmetries like the ideal observer that are based on inferences about their latent causes (i.e., the probabilistic structure of the task). Unlike the ideal observer, these models could be suboptimal by using different forms of mistuned inferences. The second class was based on heuristic principles. We used several models that more directly mapped patterns of observations, rather than observation counts, to choices. These suboptimal strategy classes gave rise to a bias-variance trade-off that is inverted relative to its typical formulation: subjects using more-complex Bayesian strategies tended to have higher bias and lower variance, whereas subjects who used less-complex heuristic strategies tended to have lower bias and higher variance. We show that these results are not predicted by the ideal observer but are a logical consequence of the different, rational ways of achieving nearly optimal task performance. The results also highlight the usefulness of breaking evidence symmetries in task paradigms aimed at studying the diversity of human inference strategies.

## Materials and methods

### Ethics statement

Human subject protocols were approved and determined to be Exempt by the University of Pennsylvania Internal Review Board (IRB protocol 844474). Subjects provided written consent on-line before they began the task.

### Experimental design

**Fig 1:**
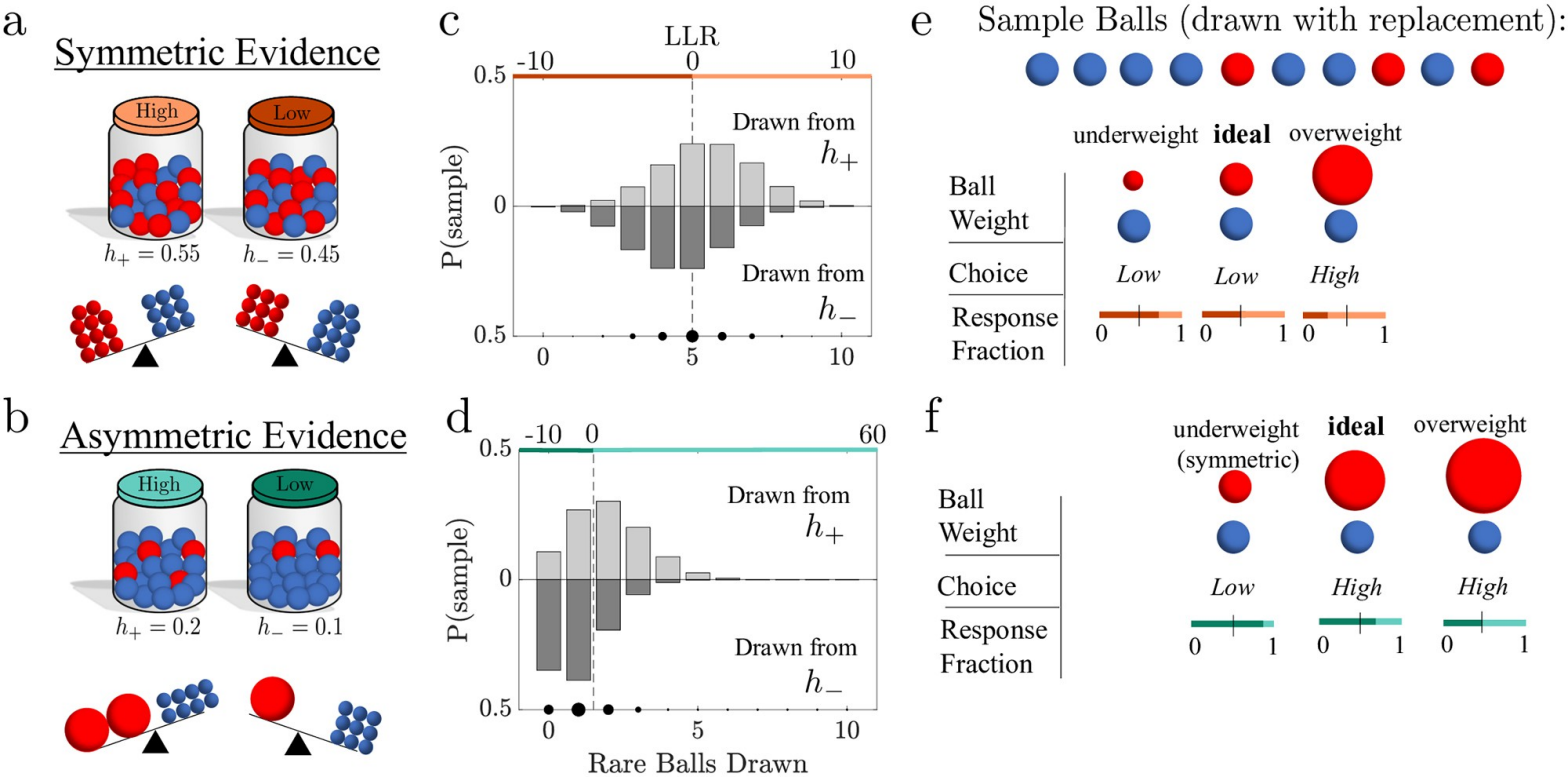
Different environmental evidence weights cause decision biases. **a-b.** Schematic of the Jar-Discrimination Task. Balls were drawn with replacement from one of two equally probable jars with different ratios of red to blue balls. Here *h*_±_ denotes the probability that a red ball is drawn from the high (*h*_+_) and low (*h*_−_) jar. We consider conditions with symmetric priors and symmetric evidence (*h*_−_ = 1 − *h*_+_; **a**), in which the red/blue ball observations had equal weights but opposite signs, or asymmetric evidence (*h*_−_ ≠ 1 − *h*_+_; **b**), in which rare (in this example red) balls were weighted more heavily in a decision. **c-d.** The corresponding probability distribution of a 10-ball sample for a given number of rare balls drawn from the high jar (*h*_+_, top) and low jar (*h*_−_, bottom) for the symmetric (**c**) and asymmetric (**d**) evidence cases. Colored bars presented on the top axis denote an ideal Bayesian observer’s jar choice resulting from the associated log likelihood ratio (LLR; an LLR of zero results in a random response). **e-f**. Example of a 10-ball sample and corresponding choices of a Bayesian observer with varying relative ball weights. **e.** Ideal ball weights for the symmetric environment produce even response fractions. **f.** Ideal asymmetric weights produce a choice asymmetry in favor of the low jar. Deviations from the ideal weights in either environment produce decision biases.

The goal of the task was to identify which of two jars was the source of a sample of balls shown to the observer. The jars were equally likely to be the source *a priori*, and subjects were informed of this fact. On each trial, subjects were shown a sample of 2, 5, or 10 red and/or blue balls drawn randomly with replacement and asked to determine which of the two jars displayed on the screen was the source of the sample (Fig 1A, S1 Fig, see Supplementary Materials S1 Text “Task and Recruitment” for additional details). The ratios of colored balls in each jar were varied to create five blocks of trials and could be described by the proportion of balls of one color, termed the “rare-ball” color. The rare-ball color remained consistent throughout all blocks. Blocks were defined by the following rare-ball fractions for the high jar (containing more rare balls)/low jar (containing fewer rare balls): Control (0.9/0.1), Hard Asymmetric (HA; 0.2/0.1), Hard Symmetric (HS; 0.55/0.45), Easy Asymmetric (EA; 0.4/0.1), Easy Symmetric (ES; 0.7/0.3).

Before beginning the full task, subjects were shown a training slideshow and performed 24 trials in the control block. To continue to the full task, each subject was required to respond correctly on at least 80% of the control trials. Subjects who did not pass this pre-test were not allowed to complete the task and were not included in our subject counts. Full sessions included randomized block orders for the remaining 4 test blocks interspersed with 12 control trials between test blocks. Subjects who achieved 50% or less on at least two of the interspersed control blocks were considered inattentive and not included in further analyses (3/ 201 subjects). Each test block consisted of 42 trials, with randomly ordered but equally sampled values of: 1) the jar used for ball draws, and 2) sample length for each trial (2, 5, or 10 balls).

**Fig 2:**
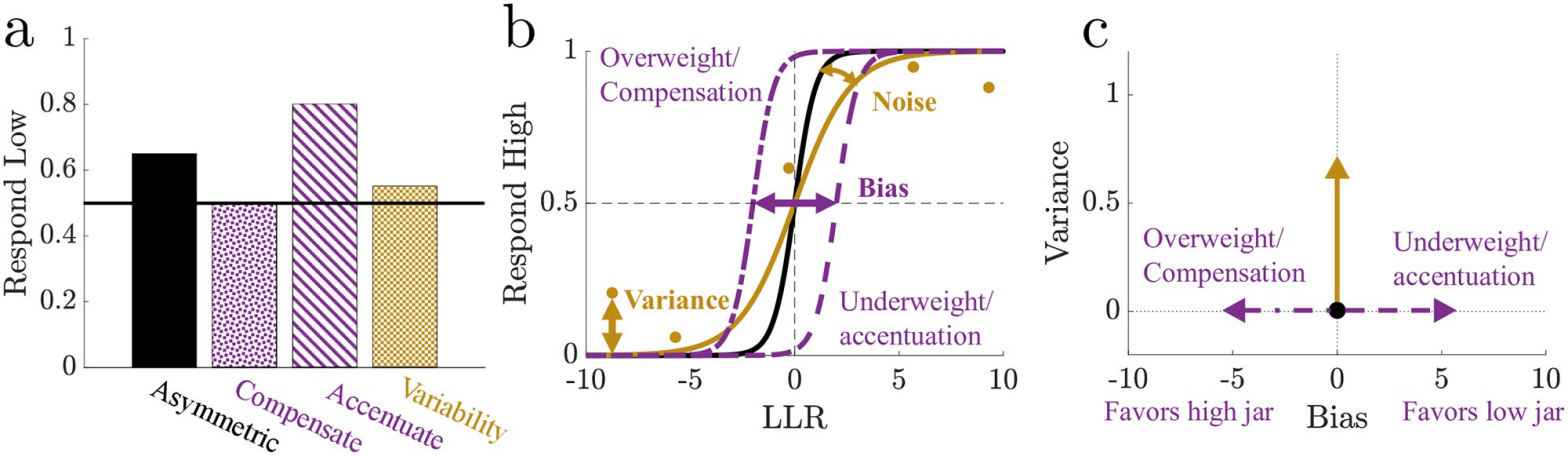
Suboptimalities are reflected by the psychometric function. **a.** Illustration of how suboptimalities, such as mistuned ball weights or biased priors, compensate for (overweighting, bias in favor of high jar) or accentuate (underweighting, bias in favor of low jar) choice asymmetry in environments with asymmetric evidence, whereas increases in variability (inclusion of noise and/or variance) have a small impact on choice asymmetry. **b**. Examples of how a psychometric function fit to data is modulated by suboptimalities. An increase in noise decreases the slope, and a bias results in a horizontal shift of the psychometric function. We define variance as the mean absolute error between the best–fit psychometric function and the data, representing systematic aspects of strategies unaccounted by the LLR. **c**. Schematized bias-variance space showing how suboptimal bias and variance shift an observer’s location in bias-variance space. Bias was bounded between [−10, 10] to mitigate overfitting due to outliers. Positive (negative) biases corresponded to more (fewer) low-jar selections.

Prior to data acquisition, we used synthetic data generated by simulating the responses from the proposed models to confirm that models were identifiable given the task conditions and could be compared to human responses given amount of data to be collected (Fig 2). We determined the number of trials in a block by balancing: 1) model parameter identifiability, with 2) reasonable task-time length for human subjects (i.e., about 30 min per session). The jar ratios were selected based on generated synthetic responses of the ideal observer, such that overall accuracy was matched between the asymmetric and symmetric blocks at each difficulty (i.e., the hard asymmetric and hard symmetric tasks were matched in accuracy). Models were developed and fit to pilot data to ensure model and parameter identifiability (See Model Fitting and Comparison below and Supplementary Materials S2 Text “Model Fitting”, S3 and S5 Figs, for more details).

**Fig 3:**
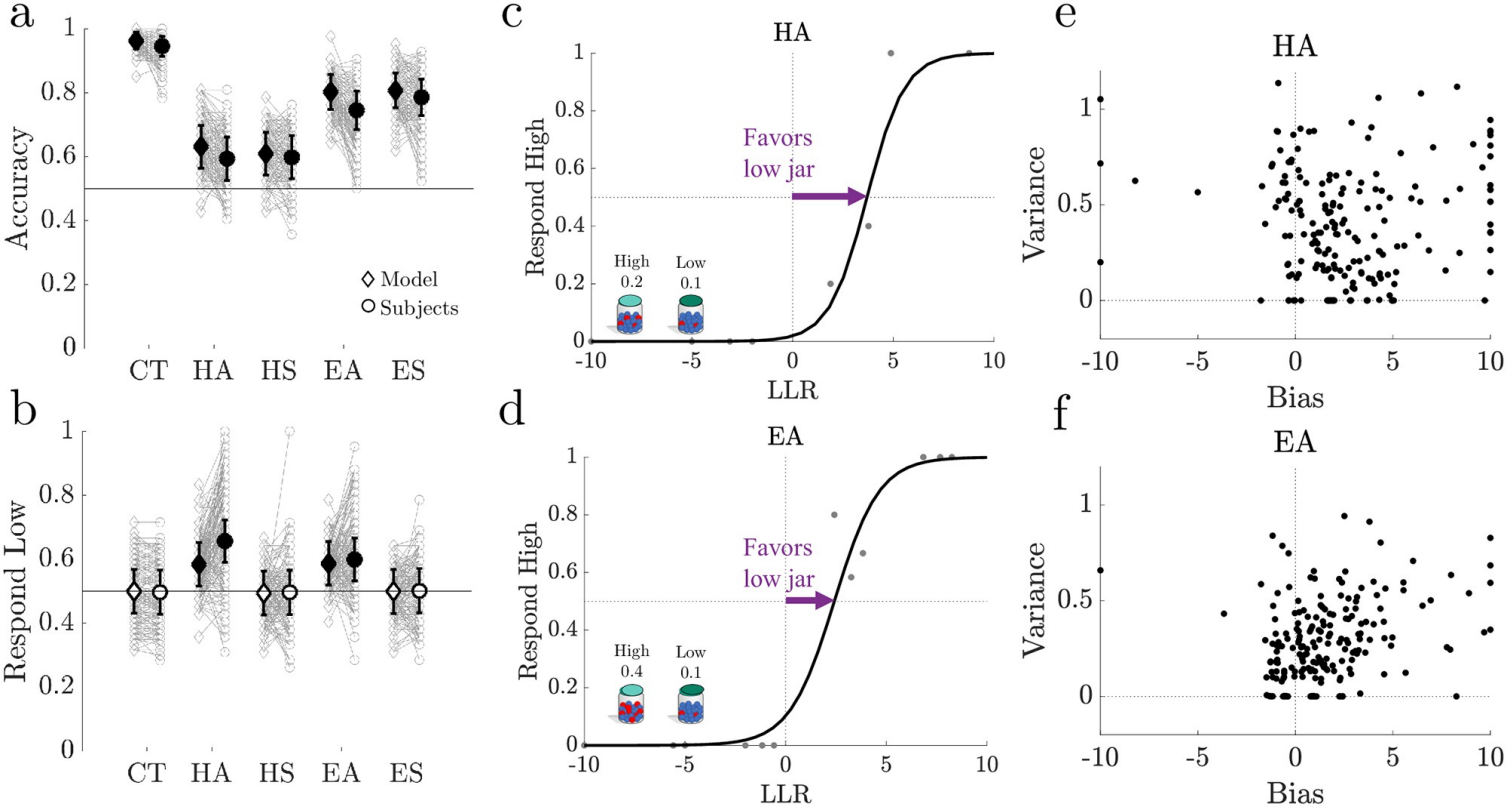
Human subjects displayed choice asymmetries that deviated from the ideal observer. **a.** Accuracy for each subject (*N* = 198, grey circles) and sample-matched ideal observer responses (grey diamonds) for each block: Control (CT), Hard Asymmetric (HA), Hard Symmetric (HS), Easy Asymmetric (EA), Easy Symmetric (ES). Population bootstrapped means (1000 iterations) and 95% confidence intervals are shown in bold. Model and subject population accuracy was significantly above chance in all cases (0.5; *p* < 0.05). **b.** Low-jar response fractions displayed as in **a**. Filled markers denote a significant population shift away from the prior (0.5; *p* < 0.05). **c-d.** Example psychometric function (line) fit to a sample subject’s high-jar responses (dots) for the HA block (**c**) and EA block (**d**) across all sample lengths. **e-f.** Bias and variance for individual subjects (points) obtained from fits of the psychometric curves to data from HA blocks (**e**) and EA blocks (**f**). Bias was bounded between [−10, 10] to mitigate overfitting to outliers. Positive (negative) biases corresponded to more (fewer) low-jar selections.

**Fig 4:**
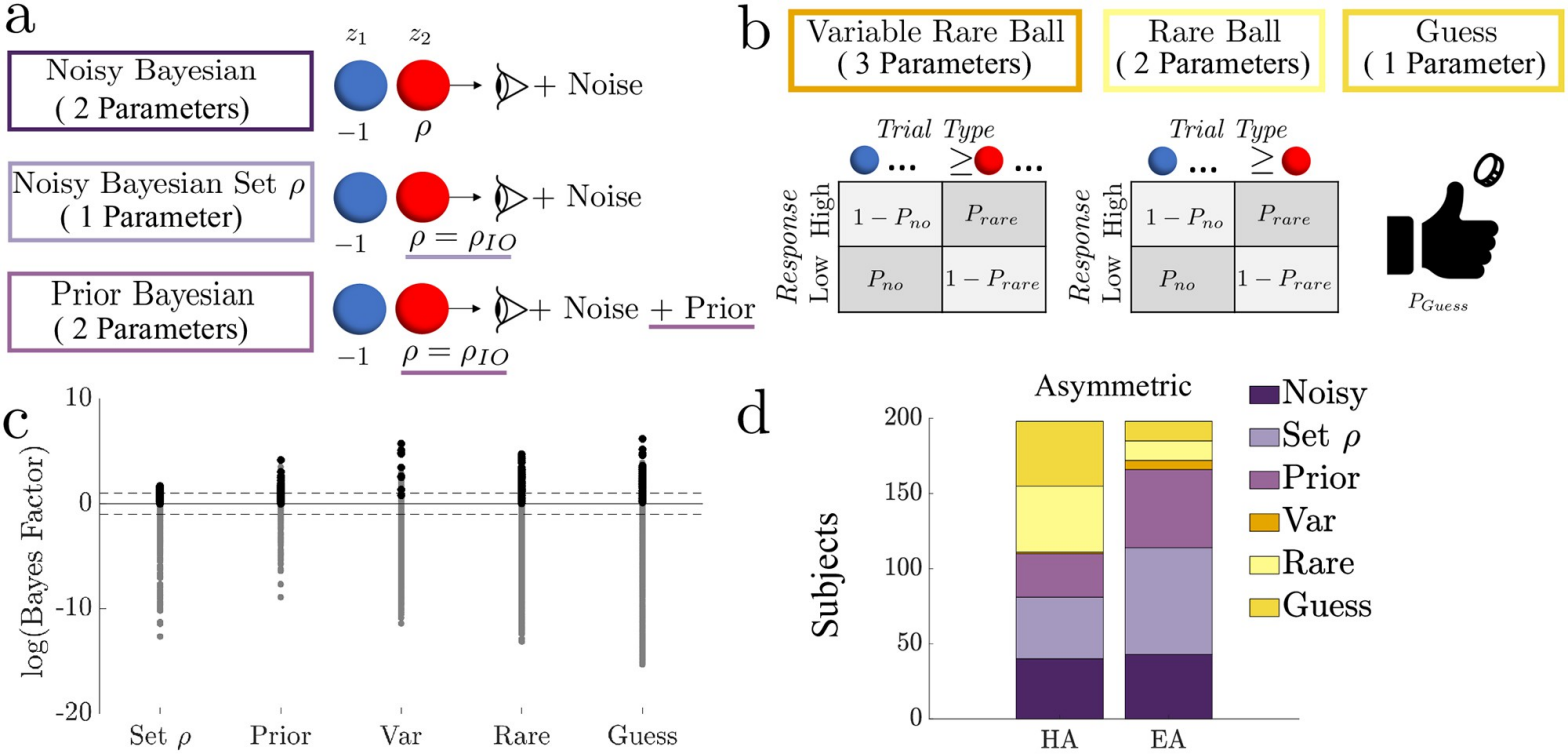
Subjects used Bayesian and heuristic strategies in asymmetric blocks. **a.** Bayesian models. Differences between the Noisy Bayesian model and alternative Bayesian models are underlined. **b.** Heuristic models. See Methods and ‘Formal model comparison’ section for more model details. **c.** Log Bayes factors (log(BF)) for each subject-block, computed between each alternative model and the Noisy Bayesian model. log(BF)>0 favors the alternative model, with log(BF)>1 or <−1 (dashed lines) providing strong evidence in favor of a given model [8]. Black (grey) markers indicate that the listed alternative model is (is not) the most likely model (percentage of subjects whose most-likely model is identified by strong evidence: 36% for Noisy Bayesian, 42% for Set *ρ*, 32% for Prior, 90% for Variable Rare, 87% for Rare Ball, 82% for Guess). **d.** Subjects categorized by the model that best describes their responses for the Hard Asymmetric (HA) and Easy Asymmetric (EA) blocks. For both blocks, a majority of the subjects’ responses were best described by Bayesian models (55% in HA, 86% in EA), but with a relatively high percentage of heuristic strategies under the HA condition.

**Fig 5:**
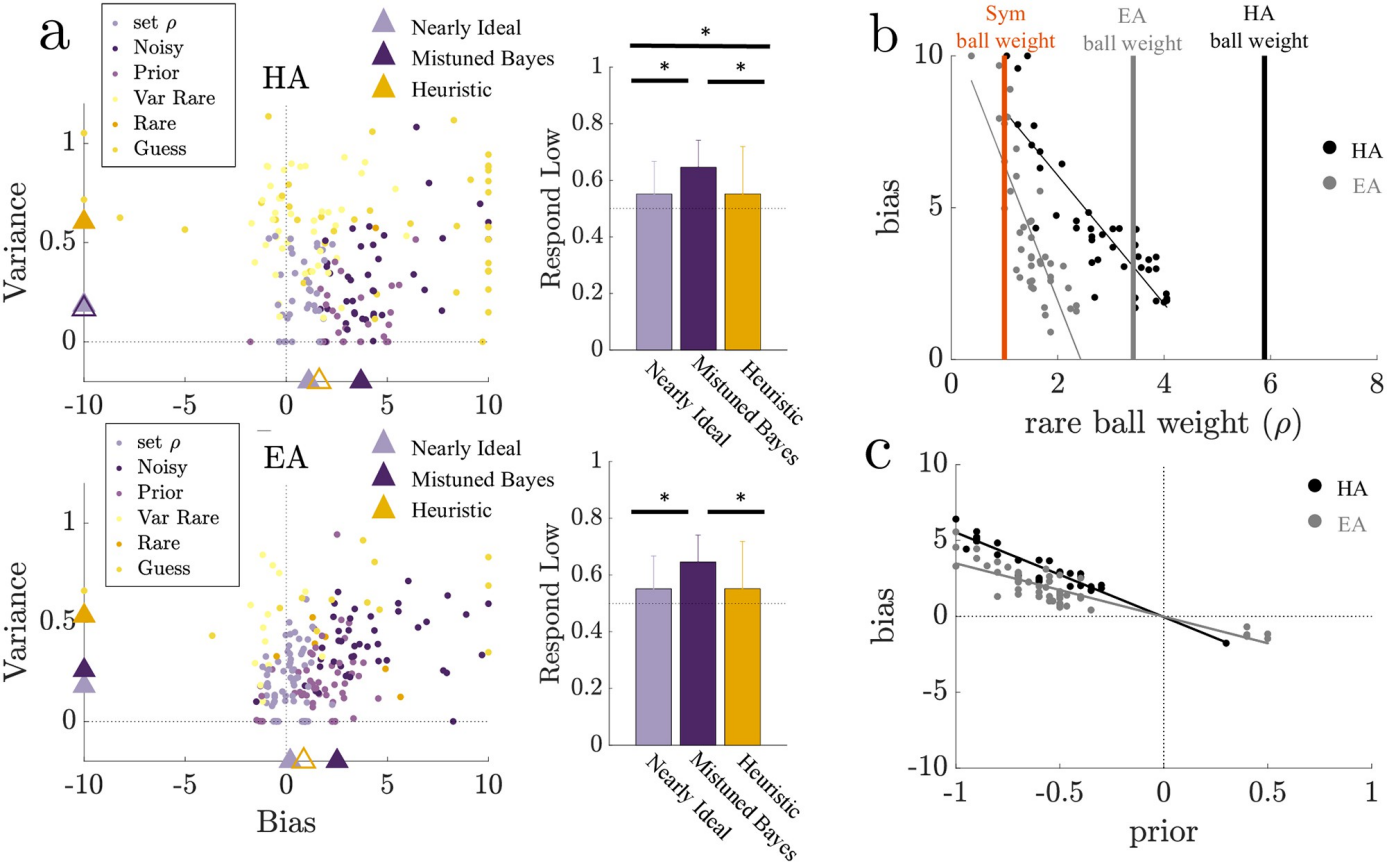
Increased bias and variance in asymmetric blocks corresponded to Bayesian subject model fits with mistuned parameters and heuristic subject model fits, respectively. **a.** Left: Hard Asymmetric (HA) and Easy Asymmetric (EA) block bias-variance plots from Fig 3E and 3F, color-coded according to each subject’s best-fitting model described in Fig 4D. Triangles denote median values for the bias-variance fits for: 1) Nearly Ideal subjects (best fit by “Noisy Bayesian Set *ρ*” model), 2) Mistuned Bayesian subjects (best fit by “Noisy Bayesian” or “Prior Bayesian” models), 3) Heuristic subjects (best fit by “Variable Rare”, “Rare Ball”, or “Guess” models). Mistuned Bayesian and Heuristic groups that significantly (not significantly) differ from the Nearly Ideal group are denoted by filled (open) triangles based on a Wilcoxon rank-sum test with *p* < 0.05. Right: Group bootstrapped means (1000 iterations) and 95% confidence intervals for low-jar responses. Statistically significant differences between groups (two-sided *t*-test with unequal variance, *p* < 0.05) are noted with an asterisk. **b.** Estimated subject bias obtained from best–fit psychometric functions compared with the maximum-likelihood estimate (MLE) of the rare-ball weight, *ρ*, for subjects best fit by the Noisy Bayesian model in asymmetric blocks (dots, EA-grey, HA-black). Regression lines are shown for group-blocks with significant correlations (Spearman correlations, *p* < 0.05). Vertical lines indicating the rare-ball weights used by the ideal observer for each asymmetric block and symmetric blocks (orange) are included for reference. **c.** Estimated subject bias from fit psychometric functions compared with the MLE of the response bias (Prior) for subjects best fit to the Prior Bayesian model in the asymmetric block (marker legend as in **b**). Negative values correspond to a bias in favor of the low jar.

We recruited 201 consenting subjects to perform the Jar-Discrimination Task on the Amazon Mechanical Turk crowdsourcing platform (95 female, 105 male, 1 non-disclosed). Subjects were recruited only if they had a 95% or better approval rating and had performed at least 100 previous approved tasks and were compensated $4.50 for completing the task. Subject location was restricted to the United States. The task and some of the analyses were preregistered at osf.io prior to data acquisition (doi: 10.17605/OSF.IO/J9XET). The preregistration described the task structure, including block length, ball samples, and type of task. Analyses presented in Figs 3A and 3B and 4, and the MLEs from Fig 5B and 5C were performed exactly as listed in the preregistration.

### Models

To develop models of the Jar-Discrimination task, we assigned the parameter *h*_±_ to refer to the proportion of rare-colored balls in a set of jars: The *h*_+_ (high) jar included more balls of the rare color, whereas the *h*_−_ (low) jar included fewer balls of the rare color, so that 0 < *h*_−_ < *h*_+_. When the proportions were symmetric, *h*_+_ = 1 − *h*_−_. When the proportions were asymmetric, 0 < *h*_−_ < *h*_+_ < 0.5.

#### Bayesian models

One class of models we considered depended on the probabilities of ball samples coming from the high or low jar that would be computed by a Bayesian observer.

#### Ideal observer

Because the two jars were always visible, we assumed the fractions of rare balls, *h*_+_ and *h*_−_, in the low and high jars are known to the ideal observer. In the simplest case without noise, an ideal Bayesian observer makes a decision based on a sample of *n* balls drawn from one of the jars, *ξ*_1:*n*_, where *ξ*_*i*_ = 1 (*ξ*_*i*_ = −1) denote an observation of a rare (common) ball color. The ideal observer uses these observations to update the log likelihood ratio (*belief*), $z_{n}=\text{log}\;\frac{P(h=h_{+}|\xi_{1:n})}{P(h=h_{-}|\xi_{1:n})}$, between the probabilities that the sample came from a jar with a rare ball frequency of *h* = *h*_+_ (high) or *h* = *h*_−_ (low). We can write the belief as:

$$\begin{array}{c} z_{n}=\sum_{j=1}^{n}\text{log}\frac{P(\xi_{j}|h_{+})}{P(\xi_{j}|h_{-})}=\sum_{j=1}^{n}\Psi \left(\xi_{j}\right), \end{array}$$

where the belief increment due to observing the color of the *j*^th^ ball is

$$\begin{array}{c} \Psi \left(\xi_{j}\right)=\text{log}\{\begin{array}{cc} h_{+}/h_{-}, & \xi_{j}=+1,\\ \left(1-h_{+}\right)/\left(1-h_{-}\right), & \xi_{j}=-1. \end{array} \end{array}$$

The most likely choice based on *n* ball draws is given by the sign of *z*_*n*_ (*z*_*n*_ > 0 ↦ choose the high jar; *z*_*n*_ < 0 ↦ choose the low jar). In all blocks, the probability that either jar was the source of the sample was 0.5, so that the ideal observer model had a flat prior, and $z_{0}=\text{log}\frac{P(h=h_{+})}{P(h=h_{-})}=0$.

In symmetric environments, *h*_+_ = 1 − *h*_−_, so

$$\begin{array}{c} \Psi \left(+1\right)=\text{log}\frac{h_{+}}{1-h_{+}}=-\frac{1-h_{+}}{h_{+}}=-\Psi \left(-1\right), \end{array}$$

and thus the magnitude of the belief increment is the same for either observation (|Ψ(+1)| = |Ψ(−1)|). When the environment is asymmetric, *h*_−_ < 1 − *h*_+_, and different ball colors correspond to different evidence weights (|Ψ(+ 1)| ≠ |Ψ(−1)|).

For *n* ball draws, we can compute the probability of the responses (choices) on a given trial, *r* = *h*_−_ and *r* = *h*_+_ for the low and high fraction jars as

$$\begin{array}{cc} P(r=h_{+}) & =P\left(r=h_{+}|h=h_{+}\right)P\left(h=h_{+}\right)+P\left(r=h_{+}|h=h_{-}\right)P\left(h=h_{-}\right)\\ P(r=h_{-}) & =P\left(r=h_{-}|h=h_{+}\right)P\left(h=h_{+}\right)+P\left(r=h_{-}|h=h_{-}\right)P\left(h=h_{-}\right) \end{array}$$

using binomial distributions. For example,

$$\begin{array}{c} P(r=h_{+})=0.5[\sum_{j=1}^{n}(\begin{array}{c} n\\ j \end{array})h_{+}^{j}h_{-}^{n-j}]. \end{array}$$

Conditioning on trial type, we can extend this analysis to obtain the minimum number of rare balls, *B*, that must be observed to produce a high jar response, given a sample of size *n*. This number is dependent on *h*_+_ and *h*_−_. When the jars are symmetric (*h*_+_ = 1 − *h*_−_), *B* = *n*/2. In asymmetric cases, *B* < *n*/2 if *h*_+_ + *h*_−_ < 1. Thus,

$$\begin{array}{c} P\left(r=h_{+}|h=h_{\pm }\right)=\sum_{k>B}^{n}(\begin{array}{c} n\\ k \end{array})h_{\pm }^{k}{\left(1-h_{\pm }\right)}^{n-k} \end{array}$$

and

$$\begin{array}{c} P\left(r=h_{-}|h=h_{\pm }\right)=\sum_{k=1}^{\left\lfloor B\right\rfloor }(\begin{array}{c} n\\ k \end{array})h_{\pm }^{k}{\left(1-h_{\pm }\right)}^{n-k}. \end{array}$$

To construct a class of Mistuned Bayesian models, we then perturbed this ideal observer model away from optimality in several ways.

#### Noisy Bayesian model

We extended the ideal observer model to include noisy belief updates, with means and variances of arbitrary magnitude. To do so we let $w_{j}∼N\left(0,a^{2}\right)$ be a normally distributed random variable with zero mean and variance *a*^2^ that was fit as a free parameter. Here we defined the belief updates by

$$\begin{array}{c} \Psi \left(\xi_{j}\right)=\text{log}\{\begin{array}{cc} \rho , & \xi_{j}=+1,\\ -1, & \xi_{j}=-1. \end{array} \end{array}$$

and

$$\begin{array}{c} z_{n}=\sum_{j=1}^{n}[\Psi \left(\xi_{j}\right)+w_{j}], \end{array}$$

where *ρ* is a free parameter representing the belief update in response to observing a rare ball, *ξ*_*n*_ = 1. Because the sign of *z*_*n*_ is all that matters for determining a model observer’s response, we normalized the update in response to a common ball to remove an unnecessary parameter. Thus, fits using this model had two free parameters: *a*^2^ and *ρ*.

#### Noisy Bayesian set *ρ* model

For this model, the belief updates are given by

$$\begin{array}{c} \rho \equiv \rho_{\text{IO}}=\frac{\text{log}\;h_{+}/h_{-}}{\text{log}\left(1-h_{+}\right)/\left(1-h_{-}\right)}, \end{array}$$

and equal to those in a rescaled ideal Bayesian model. Each belief update is perturbed additively by a Gaussian random variable with variance, *a*^2^. We set *ρ* to the optimal value *ρ*_IO_, and thus the variance, *a*^2^, was the only free parameter.

#### Prior Bayesian model

We modified the Noisy Bayesian Set *ρ* model to include a free parameter *z*_0_ for the prior. An observer using this model uses potentially unequal prior probabilities,

$$\begin{array}{c} z_{0}\equiv \text{log}\frac{\tilde{P}\left(h=h_{+}\right)}{\tilde{P}\left(h=h_{-}\right)}\neq 0, \end{array}$$

where $\tilde{P}\left(h\right)$ represents the observer’s assumed prior probability, which may differ from the true prior probability that a jar with rare ball fraction *h* is a source of the sample. A positive (negative) value of *z*_0_ implies that the observer believes *a priori* that the high (low) jar is more likely to be the source of a sample. Thus, fits using this model had two free parameters: *a*^2^ and *z*_0_.

#### Heuristic models

The other class of models (heuristic) did not depend on the likelihood functions associated with drawing a ball of a certain color from either jar.

#### Variable rare ball model

The probability of choosing either jar in the Variable Rare Ball model depends only on whether a certain number of rare balls (*θ*) are observed in a sample in the current trial (*N*),

$$\begin{array}{c} P_{\text{response}}=\{\begin{array}{c} P\left(r_{N}=h_{+}|{\text{rare}}_{\theta }\right)=P_{\text{rare}}\\ P\left(r_{N}=h_{-}|{\text{rare}}_{\theta }\right)=1-P_{\text{rare}}\\ P\left(r_{N}=h_{-}|\text{no}\;{\text{rare}}_{\theta }\right)=P_{\text{no}}\\ P\left(r_{N}=h_{+}|\text{no}\;{\text{rare}}_{\theta }\right)=1-P_{\text{no}} \end{array}\}. \end{array}$$

Here *r*_*N*_ is the response on the current trial *N*, $\left({\text{rare}}_{\theta }\right)\equiv \left(|\right|{\left[\xi_{1:n}^{N}\right]}_{+}{\left|\right|}_{1}\geq \theta )$ corresponds to observing *θ* or more rare balls (or the sum of positive entries of $\xi_{1:n}^{N}$ being at least *θ*), and $\left(\text{no}\;{\text{rare}}_{\theta }\right)\equiv \left(|\right|{\left[\xi_{1:n}^{N}\right]}_{+}{\left|\right|}_{1}<\theta )$ to observing no rare balls in the current trial (or the sum of positive entries of $\xi_{1:n}^{N}$ being less than *θ*). Thus, fits using this model had three free parameters: *θ*, *P*_rare_, and *P*_no_.

#### Rare ball

For this model we assumed that *θ* = 1, reducing the number of free parameters to two.

#### Guess model

In this model, the probability of each choice is fixed, and independent of the sample. The Guess model includes one free parameter that determines the probability of choosing the high jar:

$$\begin{array}{c} P\left(r_{N}=h_{+}\right)=P_{\text{guess}},\;\;\;\;\;\;P\left(r_{N}=h_{-}\right)=1-P_{\text{guess}}, \end{array}$$

regardless of any observations within a trial.

#### Alternative (Unused) models

In addition to the above models, we considered four alternative models, three Bayesian and one heuristic. The Bayesian models included a variation of the Noisy Bayesian with a bias in the prior probability of the two choices (3 free parameters) and a history-dependent model with asymmetry in favor of low jar responses (3 free parameters), but we found neither of these to be identifiable (see Model Fitting and Comparison below and Supplementary Materials S2 Text “Model Fitting” and S5 Fig). We also considered a windowing Bayesian model (3 free parameters), in which a specified amount of evidence was used consistently across trials (with the observer drawing from previous trials if the evidence on the current trial was insufficient), and a history-dependent rare ball model (4 free parameters), in which the probability of a choice depends on observing a rare ball in the sample, and the choice *r*_*N*−1_ on the previous trial. In both cases, fewer than 5 subjects per block were best fit by these models (Window: CT-1, HA-1, HS-3, EA-2, ES-3; Hist.-Dep: HA-1, HS-1) and were not included in further analyses. Subjects originally best fit by these models were refit with accepted models listed above, with history-dependent subjects fit by guess models and windowing subjects fit by a variety of Bayesian and heuristic strategies (7 Bayesian, 3 heuristic fits).

### Psychometric functions

We fit a a three-parameter logistic function to subject response data for each block:

$$\begin{array}{c} \rho_{b}=\alpha +\frac{1-2\alpha }{1+\text{exp}(-\beta \left(LLR_{b}-\phi \right))}. \end{array}$$

Here *LLR*_*b*_ is the true LLR of each observed set of balls as computed using the ideal observer model. We fit the following parameters: 1) *α*, the lapse rate; 2) *ϕ*, the LLR value at which each choice (high or low jar) is equally likely; and 3) *β*, the slope around the point *ϕ*. Bias was defined as a non-zero value of *ϕ*, so that positive (negative) values correspond to biases towards (away) from the low jar. Noise was defined as 1/|*β*|, so that shallower functions correspond to higher noise.

Variance was defined as the weighted average of the absolute value of the residuals (mean absolute error),

$$\begin{array}{c} v=\frac{1}{x}\sum_{i=1}^{x}n_{i}\left|P{\left(r=h_{+}\right)}_{b,i}-\rho_{b,i}\right| \end{array}$$

where *x* is the number of LLR values for a block, *n*_*i*_ is the number of trials at a given LLR value, *ρ*_*b*,*i*_ is the logistic fit for a given block-LLR, and *P*(*r* = *h*_+_)_*b*,*i*_ is the probability of a high jar response from the observer for a given block-LLR. Larger values of *v* reflected more variance.

Our interpretation is based on the idea that noise is driven by either errors in the internal representation of the LLR or post-decision choice variability, whereas variance reflects strategies that are independent of the LLR. Based on the two model classes studied here (Bayesian and Heuristic), we find that models that rely on the LLR (Bayesian models) and the subjects best fit by them are fit with some noise but substantially less variance compared to models and subjects that use a pattern-based approach that does not depend on the LLR (Heuristic models). While there is correlation between the two metrics, heuristic subjects show substantially larger values for noise, which reflect the the poor logistic fits to these responses, and the conclusions of our analyses are comparable using either metric (see Supplementary Materials S5 Text “Noise Versus Variance”, S10 and S11 Figs, for more details).

### Model fitting and comparison

#### Parameter fitting

We fit model parameters to data using Bayesian maximum-likelihood estimation. We obtained the posteriors over the parameters by considering the vectors of responses, *r*_1:42_, and observation samples, ***ξ***_1:42_, across all 42 trials in a block (***ξ***_1:60_ for the control block that had 60 trials total- 24 pre-test, 12 interspersed between each testing block). For instance, to infer the noise variance, *a*^2^, and rare-ball weight, *ρ*, in the Noisy Bayesian Model, we applied Bayes’ rule and then computed the probability of a response *r*_*N*_ in a given trial conditioned on observations ***ξ***_*N*_ as

$$\begin{array}{c} p\left(a,\rho |r_{N},\xi_{N}\right)=\frac{p\left(r_{N}|a,\rho ,\xi_{N}\right)p\left(a,\rho \right)}{p(r_{N}|\xi_{N})}. \end{array}$$

Because the denominator provides only a normalization of the probability densities of *a* and *ρ*, the primary contributions are the probability of a response *r*_*N*_ given the parameters and observations, and the prior over the parameters, *p*(*a*, *ρ*). We explain the choice of priors below. All models were defined in terms of either simple binary random variables or thresholded Gaussians, so we could evaluate the associated likelihood functions analytically. For instance, in the case of the Noisy Bayesian model, for a trial with 5 balls, and a sample containing 4 common and 1 rare ball (***ξ***_*N*_ = (−1, −1, + 1, −1, −1)), the probability of choosing the high jar, *r*_*N*_ = *h*_+_, is

$$\begin{array}{c} P\left(r_{N}=h_{+}|a,\rho ,\xi_{N}\right)=\frac{1}{\sqrt{2\pi a^{2}}}\int_{0}^{\infty }\text{exp}[-\frac{{\left(z+4-\rho \right)}^{2}}{2a^{2}}]dz=\frac{1}{2}[1-\text{erf}(\frac{4-\rho }{\sqrt{2a^{2}}})]. \end{array}$$

For models in which responses are independent across trials, we used the trial-wise response probabilities to compute the posteriors given responses and samples in a block of trials,

$$\begin{array}{c} p\left(a,\rho |r_{1:42},\xi_{1:42}\right)=\frac{p(a,\rho )}{p(r_{1:42}|\xi_{1:42})}\prod_{j=1}^{42}p\left(r_{j}|a,\rho ,\xi_{j}\right). \end{array}$$

The maximum of this posterior is the maximum likelihood estimate of the model parameters. The interval of parameters containing at least 95% of the maximum likelihood estimate were included as credible intervals for the model fits.

#### Determining model identifiability

To design the human task and determine whether the models would be identifiable from the given data, we performed model comparisons on synthetic data. We first used the Noisy Bayesian model to determine the minimum number of trials (42) needed to fit synthetic data and produce a task with a reasonable task duration for online data acquisition (30 minutes or less). However, for this model, parameters produced with a flat prior were not always identifiable, given the amount of data that we could reasonably expect to collect in a block. This problem resulted from dependencies between the noise variance, *a*^2^, and rare-ball weight, *ρ*, parameters for high values of noise.

To account for this effect, we used pilot data from 20 subjects to create an informative prior based on the subjects’ posteriors. The informative priors were computed as a smoothed version of averaged posteriors produced by the pilot subject’s fits by the Noisy Bayesian model. The averaged posterior was smoothed with respect to each parameter. To weaken the posterior with respect to *ρ*, the averaged marginal posterior was filtered using a normal distribution $N(\mu ,\sigma^{2})$ where the mean *μ* was set at the maximum value of the averaged marginal posterior and the variance *σ*^2^ was set such that the median mean squared error (MSE) of the parameter fits *ρ* for 100 synthetic Noisy Bayesian datasets was below one. The averaged marginal posterior with respect to the noise parameter *a* was smoothed using the function (*x* + *c*)/(1 + *cL*) where *x* is the marginal posterior and *c* and *L* are scaling constants selected such that the averaged posterior was smooth (no jagged edges) but did not impact the accuracy of the rare ball parameter fitting (values provided in S4 Fig). Given that a low-noise parameter *a* was identified for most pilot subjects and that higher values of *a* could correspond with underweighting the value of *ρ*, we prioritized accurately identifying *ρ* (see Supplementary Materials S2 Text “Model Fitting” and S3 Fig for more details).

To confirm that the new informative priors produced realistic fits for all of our Bayesian models, we applied the informative priors to model fits for data of 100 synthetically generated datasets with randomly selected model parameters for each Bayesian model per block and found that the credible intervals contained the true parameters for the parameter values predicted by the informative prior. The fits to the synthetic datasets also matched the averaged posteriors from the pilot data, with a strong preference for low-noise parameter values and values at or below the true rare-ball weight. Thus, using informative priors did limit identifiability at high noise variance and rare-ball weight values, but we ensured that our models could be correctly identified near or below the values predicted by the ideal observer, as was suggested by the pilot data. The true parameters from synthetic datasets from the heuristic models with a flat prior were also recoverable with low MSE.

We then generated model responses from 100 randomly sampled versions of each candidate model (sampling from the informative priors for Bayesian-based models and flat priors for heuristics) to confirm that each model could be appropriately selected when compared to other models. We performed model comparison and selection using log Bayes factors (log(BF)), comparing the likelihood that a particular dataset came from one of two models by computing the log likelihood ratio of the marginal likelihoods for any given pair of models,

$$\begin{array}{c} \text{log}\left(\text{BF}\right)=\text{log}\frac{P(D|M_{2})}{P(D|M_{1})}=\text{log}\frac{P\left(M_{2}|D\right)P\left(M_{1}\right)}{P\left(M_{1}|D\right)P\left(M_{2}\right)}. \end{array}$$

Here *D* is the data from a block of trials (*r*_1:42_ and ***ξ***_1:42_), and *M*_1_ and *M*_2_ are two models from the list we described above. For example, to compare the Noisy Bayesian model to the Prior Bayesian model for a given block, we integrate the probability of responses conditioned on observations and parameters against the priors over the model parameters:

$$\begin{array}{c} \text{log}\;\text{BF}=\text{log}\frac{\int_{-\infty }^{\infty }\int_{0}^{\infty }p\left(r_{1:42}|a,z_{0},\xi_{1:42}\right)p\left(a,z_{0}\right)dadz_{0}}{\int_{0}^{\infty }\int_{0}^{\infty }p\left(r_{1:42}|a,\rho ,\xi_{1:42}\right)p\left(a,\rho \right)dad\rho }. \end{array}$$

For all comparisons, we used the Noisy Bayesian model as the baseline model (in the denominator of the Bayes factor); i.e., model 1 (*M*_1_). We found that two candidate models were not identifiable as listed above (one assumed an asymmetric repetition bias, the other included a biased prior and free parameter for rare-ball weight; Bayes factors correctly selected the true model < 80% of the time) and thus were excluded from our analyses (see Supplementary Materials S2 Text “Model Fitting”, S5 Fig, for additional details and analyses).

#### Subject model selection

To determine the model that best described a human subject’s responses on a particular block, we computed the log Bayes factors between each alternative model and the Noisy Bayesian model. Positive values of the log Bayes factor provided evidence in favor of a particular alternative model over the Noisy Bayesian model, with evidence growing with the magnitude of the factor (we chose |log BF| > 1 to indicate strong evidence in favor of a model [8]). The most-likely model was selected based on the maximal log Bayes factor value across all alternative models. If no values were >0, the Noisy Bayesian model was selected.

#### Subject cross-validation

For each block and subject, we used 10-fold 90/10 cross-validation to test the predictive power of the model identified using Bayes factors that best describes the subject’s responses. To do so, we fit the model to data from 90% of the trials from the block and used the result to predict the subjects’ responses on the final 10% of trials in the block. We repeated this process 10 times and computed the accuracy of the model by comparing its predictions to the subject’s responses and averaging across all 10 iterations. (See Supplementary Materials S3 Text and S8 Fig for details.)

### Rate-distortion theory

We applied rate-distortion theory to compare the subjects’ accuracy (fraction correct) to the maximal accuracy bound obtainable by an ideal observer constrained to a fixed amount of mutual information (MI) between an observer’s response, *r* and the observation on a trial. We describe this observation as a random variable (|*ξ*|, *n*), where *n* is the size of a sample, and |*ξ*| is the number of rare balls in the sample, as:

$$\begin{array}{c} I_{B}^{i}\left(|\xi |,n;r\right)=\sum_{n\in \{2,5,10\}}\sum_{\left|\xi |\in \{0,\ldots ,n\right\}}\sum_{r=h_{\pm }}P_{B}^{i}\left(|\xi |,n,r\right){\text{log}}_{2}\frac{P_{B}^{i}\left(|\xi |,n,r\right)}{P_{B}^{i}\left(|\xi |,n\right)P_{B}^{i}\left(r\right)}, \end{array}$$

where *i* is a subject or model, *B* is the block. We computed the probabilities $P_{B}^{i}$ empirically.

To obtain subject estimates we used all response and observation data for the 42 trials within a block, so any particular observation sample not seen was not included in the sum. Each subject’s trials within a block were bootstrapped by uniformly resampling the data 1000 times to obtain a distribution of MI and accuracy estimates for the block.

The MI with the inclusion of the previous trial was defined as:

$$\begin{array}{c} I_{B}^{i}(|\xi |,n,r_{-1},r)=\sum_{n,|\xi |,r_{-1},r}P_{B}^{i}(|\xi |,n,r_{-1},r){\text{log}}_{2}\frac{P_{B}^{i}(|\xi |,n,r_{-1},r)}{P_{B}^{i}(|\xi |,n,r_{-1})P_{B}^{i}\left(r\right)}, \end{array}$$

where the sums are taken over ball counts *n* ∈ {2, 5, 10}, number of rare balls *ξ* ∈ {0, 1, …, *n*}, and previous *r*_−1_ ∈ *h*_±_ and current *r* ∈ *h*_±_ trial response.

To define the accuracy bound for an optimal observer, we computed MI in the limit of many samples, allowing for a calculation directly using probability mass functions. As such, we considered all possible samples $\xi_{B}^{i}$, disregarding ball order, (*n* + 1 possible counts for trials with *n* = 2, 5, 10 ball draws) in Ξ and responses $r_{B}^{i}$ in *R*:

$$\begin{array}{c} I_{\pi }\left(\xi_{B}^{i},r_{B}^{i}\right)=\sum_{\xi_{B}^{i}\in \Xi }P\left(\xi_{B}^{i}\right)\sum_{r_{B}^{i}\in R}\pi \left(r_{B}^{i}|\xi_{B}^{i}\right){\text{log}}_{2}\frac{\pi (r_{B}^{i}|\xi_{B}^{i})}{p(r_{B}^{i})}, \end{array}$$

where $\pi (r_{B}^{i}|\xi_{B}^{i})$ is the policy used to generate responses from observations across the block. Note, that this is simply given by the standard ideal observer model defined above when fixing the MI to unity. However, for values of MI less than one, we employed an optimization procedure, which we describe below, in order to obtain the optimal policy that uses a fixed MI budget.

#### Computing the optimal bound

The rate-distortion bound can be computed according to a constrained-optimization problem in which we identify the maximum possible accuracy for a given level of MI in the limit of many trials. In the ideal observer case, the policy applied to compute MI and accuracy is:

$$\begin{array}{c} \pi \left(r=h_{+}|\xi^{c}\right)=\{\begin{array}{cc} 1, & \xi^{c}>B,\\ 0.5, & \xi^{c}=B,\\ 0, & \xi^{c}<B. \end{array} \end{array}$$

(and *π*(*r* = *h*_−_|*ξ*^*c*^) = 1 − *π*(*r* = *h*_+_|*ξ*^*c*^)) where *ξ*^*c*^ ∈ {0, 1, 2, …, *n*} is the count of rare balls observed and *B* is the number of rare balls required to trigger a high jar response. Note this provides a specific accuracy bound for a fixed value of MI, corresponding to the ideal observer. Additionally, we must compute the predictive accuracy using the value function applied to a particular policy *π*

$$\begin{array}{c} V_{\pi }=\sum_{\xi^{c}}p\left(\xi^{c}\right)\sum_{r\in \{h_{+},h_{-}\}}\pi \left(r|\xi^{c}\right)Q\left(\xi^{c},r\right), \end{array}$$

which sums over all possible combinations of unordered sample counts (*ξ*^*c*^ = 0, 1, 2, …, *n* rare balls for *n* = 2, 5, 10 balls in a trial) for which we can always compute the trial specific value function from the ideal observer $Q\left(\xi^{c},r\right)=1/\left(1+e^{-z_{n}}\right)$, where *z*_*n*_ is the ideal observer’s log likelihood ratio.

Thus, to bound accuracy for a given MI (*I*_*π*_ ≡ *C*), we maximized the value function according to the best policy that uses the prescribed MI:

$$\begin{array}{c} V^{*}=\underset{\pi }{\text{max}}V_{\pi }\;\;\;s.t.\;\;\;I_{\pi }\equiv C, \end{array}$$

which generates the optimal predicted bounds. This maximization problem was solved using MATLAB’s constrained optimization package (fmincon) with a constraint given by *I*_*π*_ ≡ *C* and *V*_*π*_ as the objective function.

### Algorithmic complexity

As in [9], algorithmic complexity is described by the number of operations required for each strategy, broken into 4 types: 1) arithmetic (*A*), 2) written into memory (*W*), 3) stored in memory (*S*), 4) read from memory (*R*). Thus complexity is defined as

$$\begin{array}{c} C=C_{reflex}+\left\langle N^{A}\right\rangle +\left\langle N^{W}\right\rangle +\left\langle N^{S}\right\rangle +\left\langle N^{R}\right\rangle \end{array}$$

where *C*_*reflex*_ is the reflexive cost, constant across models. 〈*N*^*i*^〉 are the ${\text{lim}}_{T\to \infty }\frac{1}{T}\sum_{t=1}^{T}N_{t}^{i}$ for each operator type. For each model, the number of operations are summed to compute the algorithmic complexity. Details on the operations counted for each strategy used here are found in S16 Fig and described in Supplementary Materials S7 Text “Complexity Analyses”. For the Bayesian models, operations scaled with the number of balls in the sample, while heuristic models defined one value for algorithmic complexity across all sample lengths.

### Statistics

Population statistics were computed by uniformly bootstrapping 1000 times from each data set, using the same number of samples as the original sample, to identify the mean and confidence intervals.

Correlations were computed using Spearman’s correlation. Differences between medians were computed using a two-sided Wilcoxon rank-sum test. We defined significance as *p* < 0.05.

## Results

We used a form of a classic inference task that required each subject to infer which of two *a priori* equally likely jars filled with red and blue balls was the source of a sample of balls drawn with replacement (Fig 1A). On each trial, the sample of 2, 5, or 10 balls was shown all at once, with the contents of both jars visible at all times, and it was known that each jar was equally likely to be the source on each trial. Across different blocks, the proportions of red and blue balls in each jar were varied, thereby altering the ideal evidence weight of each observation. Under “symmetric” conditions, the ratios of the two ball colors in the two jars were reciprocal, such that the rare color in one jar was the common color in the other. In contrast, under “asymmetric” conditions, the ratios were non-reciprocal, such that both had the same rare (and common) color, but in different proportions. The jar with more rare balls was termed the “high” jar, and the jar with fewer rare balls was termed the “low” jar. We asked how optimal, suboptimal, and human observers compare in their use of symmetric and asymmetric information to infer the jar source (see Supplementary Materials S1 Text “Task and Recruitment” and S1 Fig for more details on the task structure).

### Optimal inference

We first derived the strategy of an ideal Bayesian observer that optimizes accuracy given the known task structure. Because the two jars are always visible, the ideal observer knew the fraction of rare balls in each jar *h*_±_, where *h*_+_ described the rare ball fraction in the high jar and *h*_−_ corresponded to the low jar so that 0 < *h*_−_ < *h*_+_. When the proportions were symmetric, *h*_+_ = 1 − *h*_−_, so rare/common balls were weighted equally. When the proportions were asymmetric, 0 < *h*_−_ < *h*_+_ < 0.5, so rare balls were weighted more heavily than common balls (Fig 1A and 1B).

The ideal observer saw a sample of ball draws all at once, *ξ*_1:*n*_, where *ξ*_*i*_ = 1(*ξ*_*i*_ = −1) if a rare (common) ball was drawn, and computed the *belief* as the log-likelihood ratio (LLR), $z_{n}=\text{log}\frac{P(h_{+}|\xi_{1:n})}{P(h_{-}|\xi_{1:n})}$, between the probabilities that the sample of draws came from either jar. When jar proportions were symmetric, the ideal observer considered only the fraction of rare (or, equivalently, common) balls sampled to determine the more likely jar. When jar proportions were asymmetric, rare balls provided more evidence than common ones. The more likely jar given *n* observations was determined by the sign of *z*_*n*_: *z*_*n*_ > 0 ↦ choose the high jar; *z*_*n*_ < 0 ↦ choose the low jar.

The impact of evidence asymmetry on ideal-observer choices could be illustrated by comparing the probability distributions of rare balls in a 10-ball sample. For symmetric jars, the distributions of rare-ball counts was symmetric about the midline, at 5 observed rare balls (Fig 1C). Thus, the ideal observer’s beliefs and choices were also symmetric in this environment, and they were both consistent with the prior (Fig 1E). In contrast, asymmetric jars produced rare-ball distributions that were skewed based on the *h* values. For the asymmetric example shown, counts of zero or one rare ball(s), which corresponded with the ideal observer choosing the low jar, occurred more often than counts of two or more rare balls, which corresponded with the ideal observer choosing the high jar (Fig 1D). Thus, in the asymmetric case, the appropriate weighting of evidence by the ideal observer led to a choice asymmetry in favor of low-jar choices, even when using the correct prior (Fig 1F).

### Suboptimal inference

To identify suboptimalities in the performance of both simulated and human subjects for this task (Fig 2A), we analyzed choice data in terms of psychometric functions that related the fraction of high-jar choices to the observed LLR (Fig 2B). For an ideal observer, this relationship was a step function, with the step at LLR = 0, regardless of the asymmetry of choice fractions. For real and simulated data, we fit choice probabilities to a logistic function. We defined bias as the horizontal shift of the best–fit logistic function, so that positive (negative) shifts correspond to biases that accentuate (compensate for) choice asymmetry. We decomposed choice variability into two components: 1) noise, which we assumed was purely stochastic and therefore did not depend on specific patterns of observations, defined as the inverse of the slope of the logistic function, so that shallower functions corresponded to higher noise; and 2) variance, which we assumed was sensitive to specific observations that were not accounted for by the LLR-dependent psychometric function (i.e., different combinations of balls that correspond to the same LLR might lead to systematically different choice patterns), defined as the mean absolute error between the data and the best–fit logistic function. Below, we focus on variance (Fig 2C) but include comparable analyses of noise in Supplementary Materials S5 Text “Noise Versus Variance”, which showed that noise and variance were correlated with each other (S10 Fig) and our conclusions were consistent with both metrics (S11 Fig).

### Human behavior

We used the crowdsourcing platform Amazon Mechanical Turk (MTurk) to recruit 201 subjects to perform the Jar-Discrimination task (Fig 1A). Each subject first performed 24 relatively easy control (CT, *h*_+_ = 0.9/*h*_−_ = 0.1) trials with symmetric jars, and then performed 42 trials under each of four testing conditions that varied in difficulty and evidence asymmetry: Hard Asymmetric (HA, *h*_+_ = 0.2/*h*_−_ = 0.1), Hard Symmetric (HS, *h*_+_ = 0.55/*h*_−_ = 0.45), Easy Asymmetric (EA, *h*_+_ = 0.4/*h*_−_ = 0.1), and Easy Symmetric (ES, *h*_+_ = 0.7/*h*_−_ = 0.3). Subjects were told that each jar was equally likely to be the source on each trial, and the contents of both jars visible at all times. Details about the task structure, including task pre-registration, and subject participation can be found in the Methods and Supplementary Materials S1 Text“Task and Recruitment.” (S1 and S2 Figs) For simplicity, we have included results from symmetric and asymmetric blocks in Fig 3A and 3B but focus on asymmetric blocks in the remainder of the manuscript. Results from symmetric blocks can be found in Supplementary Materials S6 Text “Symmetric Results”, for comparison purposes (S12 Fig).

Overall, the subjects’ accuracy tended to be above chance (bootstrapped means and 95% confidence intervals were significantly above 0.5 for population data from each of the five blocks) and in many cases was qualitatively similar to that of the ideal observer under matched conditions (Fig 3A). Moreover, for asymmetric conditions both the ideal observer and the subjects had choice asymmetries in favor of the low jar that deviated from the prior (Fig 3B, bootstrapped means and 95% confidence intervals of low-jar responses significantly above 0.5).

However, the subjects also exhibited numerous suboptimalities in the asymmetric blocks. These suboptimalities included errors attributable to bias and variance (Fig 3C and 3D) that varied in magnitude across individual subjects but, in general, were larger than expected, given the responses of the ideal observer (Fig 3E and 3F). Although bias varied in magnitude and sign, most cases corresponded to an accentuation of choice asymmetry favoring the low jar. Likewise, variance ranged from zero, corresponding to choices that exactly matched the best-fitting logistic psychometric function, to near one, corresponding to choice patterns that deviated substantially from the best-fitting psychometric function. These effects were amplified by short sample lengths and task difficulty (see Supplementary Materials S4 Text “Choice-Asymmetry Analyses” and S9 Fig for details).

### Formal model comparison

To relate these human behavioral patterns to particular inference strategies, we fit Bayesian-based and heuristic models separately to each individual subject’s responses per block. We used Bayes factors to select the model that best matched each subject’s responses on a given block and further confirmed the fits by cross-validating the subject responses with the best-fit model (S8 Fig). We then determined the bias-variance trends for each subject’s best-fitting model based on the subjects’ psychometric fits (details on model selection and fitting can be found in the Methods and Supplementary Materials S2 Text “Model Fitting” and S3 Text “Subject Model Fitting”, S6 and S7 Figs).

Three models we used were Bayesian-based (Fig 4A). The first model assumed that the observer makes decisions based on a noisy version of the log-likelihood, in which noise was a normally distributed random variable with zero mean and a free parameter for variance, and *ρ* was a free parameter representing the belief update in response to observing a rare ball (“Noisy Bayesian”). When *ρ* > 1, the model weighted a rare-ball observation more strongly than an observation of a common ball. For the second model, we set *ρ* to the ideal observer’s rare-ball weight. Without noise, this version is equivalent to the ideal-observer model (“Noisy Bayesian Set *ρ*”). In the third model, we added a parameterized prior to the “Noisy Bayesian set *ρ*” model (“Prior Bayesian”). Together these models allowed us to identify subjects whose choices were consistent with principles of Bayesian inference but possibly corrupted by suboptimalities associated with belief noise, rare-ball mis-weighting, and/or an inappropriate prior.

Three other models we considered were heuristic strategies that, unlike Bayesian-based observers, assumed that decisions were not based on likelihoods but rather specific patterns of observed balls (Fig 4B). The first model assumed that the probability of choosing the high jar, *P*_rare_, is determined by whether the number of observed rare balls exceeded a threshold. This threshold was a model parameter whose value we inferred from subject responses (“Variable Rare Ball”). Because the threshold was fixed regardless of the total ball count (2, 5, or 10), the model could produce different response probabilities for different ball patterns with the same LLR. The second model was a reduction of the Variable Rare Ball model based on the assumption that the observer chooses the high jar with some probability whenever one or more rare balls are observed (“Rare Ball”). This assumption is equivalent to fixing the threshold parameter in the Variable Rare Ball model to 1. The third model described a simple guessing strategy (“Guess”), in which the observer selected the high jar with a probability that was fixed across trials (and thus did not depend on the specific observations on a given trial) but could produce an overall bias when its value differed from 0.5.

We determined whether each subject’s responses were better described by either a Bayesian or heuristic strategy by computing Bayes factors between the Noisy Bayesian and alternative models (Fig 4C). Most subjects exhibited choice behaviors that were most consistent with one of the Bayesian models (Fig 4D, > 50% of subjects per block), although the hard asymmetric block showed the highest percentage of subjects identified as using heuristic strategies. Of subjects best described by a heuristic model, a majority (82–90% in each block) had Bayes factors that provided strong evidence in favor of the heuristic model (i.e., log(*BF*) > 1 [8]; Fig 4C).

### Model-dependent bias-variance trends

There was a systematic relationship between the model that best described a subject’s responses and the magnitude of their bias and variance as determined by their best–fit psychometric function (Fig 5A). Specifically, responses of subjects best described by a nearly ideal Bayesian model (i.e., the Noisy Bayesian Set *ρ* model, referred to as the “Nearly Ideal” group) were characterized by almost no bias and small variances. The choice asymmetries of these subjects were similar to those of the ideal observer. The remaining subjects exhibited suboptimalities that differed depending on whether the subject’s choices were best described by a heuristic or a Bayesian-like model. Suboptimal Bayesian-like models that described subject’s choices were “mistuned” versions of the ideal observer, which performed the same computation as the ideal observer but with parameter values (e.g., rare ball weight *ρ*) that did not match the optimal parameter value. The median of the bias parameter from the group of subjects best described by heuristic models (referred to as the “Heuristic” group) was close to zero, but but the median of the variance parameter for this group was relatively high for both of the asymmetric conditions. In contrast, the median variance for the group of subjects best described by suboptimal Bayesian-like models (i.e., the Noisy Bayesian or Prior Bayesian model, referred to as the “Mistuned Bayesian” group) was low, but the group showed high median bias in favor of the low jar, which resulted in a significantly larger low-jar response fraction than either the Nearly Ideal or Heuristic groups (Fig 5A, right plots; two-sided *t*-test with unequal variance, *p* < 0.05).

Thus, the Mistuned Bayesian group differed in their bias and the Heuristic group differed in variance from the Nearly Ideal group (Wilcoxon rank-sum, *p* < 0.05). Moreover, the relatively high biases exhibited by the Mistuned Bayesian group reflected a mistuning of LLR-relevant parameters. For subjects best fit by the Noisy Bayesian model, this mistuning involved the weight of evidence from rare-ball observations, *ρ*, which was underweighted compared to the ideal observer, particularly in the hard asymmetric block (Fig 5B). For subjects best fit by the Prior Bayesian model, this mistuning involved the prior, which was biased and most often favored the low jar (Fig 5C; Spearman correlations, *p* < 0.05). In contrast, the relatively high variance exhibited by the Heuristic group was attributed to choice independence from the LLR, with strategies that did not accumulate weighted evidence like the Bayesian models.

### Complexity-dependent bias-variance trends

**Fig 6:**
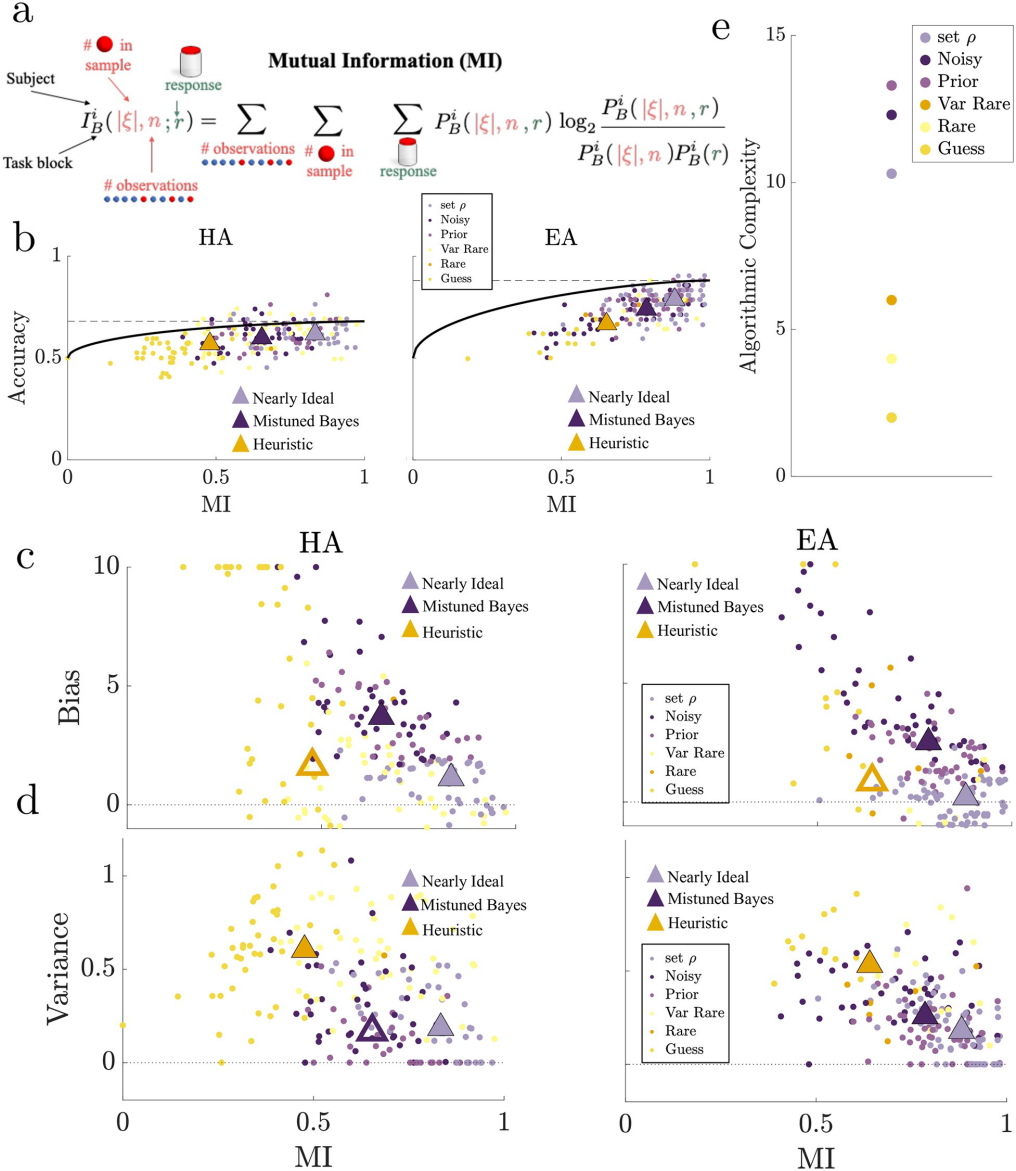
More complex but suboptimal human strategies exhibited more bias. **a.** Mutual information (MI) between the number of rare balls in a sample (|*ξ*|), the sample length (*n*), and the response (*r*) for each subject and block. **b.** Accuracy versus MI (computed as bootstrapped means from 1000 iterations per subject) for the Hard Asymmetric (HA) and Easy Asymmetric (EA) blocks. Dots represent data from individual subjects, color coded by subject’s best-fitting model described in Fig 4D. Black line represents the accuracy bound (the maximum accuracy attainable by the idea observer for a fixed MI in the limit of many trials). The dashed horizontal lines indicate the accuracy bound for maximum MI values. Note that points could exceed the asymptotic accuracy bound because the number of trials for each subject was finite. Median values for the Nearly Ideal, Mistuned Bayesian and Heuristic subject groups are indicated with triangles. In each case, filled Mistuned Bayesian and Heuristic triangles denote statistically significant differences in MI from the nearly ideal group (*p* < 0.05) based on a Wilcoxon rank-sum test. Median values for all 3 groups showed increase in both accuracy and MI ranking from lowest (Heuristic), middle (Mistuned Bayesian), highest (Nearly Ideal). **c- d.** Relationship between estimated bias (**c**) and variance (**d**) from the fit psychometric function for each subject and MI, triangles represented as in **b** based on statistically significant differences in bias or variance. **e.** Algorithmic complexity for each model. Bayesian models shown as the mean algorithmic complexity across sample lengths.

To understand how bias and variance were related to the complexity of the strategies the subjects employed on a task, we used two complementary approaches to quantify strategic complexity. The first approach was purely data-driven, allowing us to avoid making assumptions about the specific, algorithmic form of each strategy. This approach was based on the idea that efficient inference strategies solve an “information bottleneck” problem [10], which is closely related to lossy data compression and rate-distortion theory [11]; i.e., maximizing predictive accuracy for a fixed information budget. Specifically, for this approach we computed two quantities using data separately from each subject and block: 1) strategic complexity, measured as the mutual information (MI) between the subject’s observations (the samples of balls observed on each trial) and their choices in the given block (Fig 6A), where larger values implied that the known ball sample reduced uncertainty in a subject’s choice; and 2) strategic effectiveness, measured as the proximity of the subject’s accuracy to the maximum achievable accuracy given their strategic complexity (termed the “optimal accuracy bound”; for details see the “Complexity Analyses” S7 Text of the Supplemental Materials), where smaller values implied that the strategy was being used more effectively to generate correct choices for a given level of complexity. Note, high complexity does not necessarily imply high accuracy since complex strategies could use irrelevant information and/or be ineffective, increasing the distance to the maximal achievable accuracy.

In general, subjects who used more-complex strategies (i.e., those who used more information from the current trial to make choices) were more accurate, with subjects that used the most-complex strategies most closely approaching the optimal accuracy bound (i.e., they used information more effectively) (Fig 6B). Moreover, both accuracy (absolute accuracy and proximity to the accuracy bound) and complexity depended systematically on strategy type, with the responses of Heuristic subjects characterized by the lowest MI and accuracy, responses of Mistuned Bayesian subjects showing increased MI and accuracy, and responses of Nearly Ideal subjects being the most complex and accurate (Fig 6B). Given the increases in complexity from Heuristic to Mistuned Bayesian to Nearly Ideal subjects (Wilcoxon rank-sum test, *p* < 0.05), it followed that subjects that used suboptimal strategies in the asymmetric conditions exhibited a bias-variance trade-off that was inverted relative to its typical formulation: the less-complex Heuristic subject group tended to make errors characterized by higher variance but lower bias, whereas the more complex but suboptimal Mistuned Bayesian subject group tended to make errors characterized by lower variance but higher bias as compared to the most-complex Nearly Ideal subject group (Fig 6C and 6D, Wilcoxon rank-sum *p* < 0.05). This complexity-based ordering of strategies, from simpler heuristics to more complex Bayesian-based strategies, was robust to an alternative MI metric that included the subject’s choice from the previous trial as a source of irrelevant information. These trends relating model complexity, bias, and variance were also apparent in simulated model data using distributions of parameter values that mimicked the subject fits from each group. Moreover, within model groups, complexity, bias, and variance were correlated, with bias and variance increasing as MI decreased, reinforcing that observed inversion of the bias-variance trade-off corresponded with differences in overall strategy type (further details about these alternative measures can be found in Supplementary Materials S7 Text “Complexity Analyses”, S13, S14 and S15 Figs).

The second approach we used to quantify strategic complexity was based on the algorithmic complexity of the best–fitting model for a given subject in the given block. This metric is useful for quantifying the capacity of an algorithm to perform multiple operations that could, in principle, affect performance flexibility [9, 12–14]. Moreover, it can provide insights into strategic complexity beyond simpler quantities like the number of free parameters (which was similar for many of our models; see Fig 4) that accounts for part of a given model’s ability to process information in a flexible (complex) manner [15]. Specifically, algorithmic complexity assigns computational costs to each component of the strategy by counting the total number of operations (arithmetic, writing to memory, reading from memory, and storage) needed to perform the task. Based on our assignments, this metric showed a sample-length dependent scaling in Bayesian complexity, but still confirmed that measures of complexity for the Bayesian models were much larger than those of heuristics (Fig 6E). These model-based results support the idea that the observed patterns of bias and variance are inherent to the relationship between the strategies described by these models and not simply idiosyncrasies of the subjects’ behavioral patterns, with errors in more-complex Bayesian-like strategies leading to increased biases, but less-complex strategies based on the pattern of observations leading to increased variance (details of this analysis can be found in Supplementary Materials S7 Text “Complexity Analyses”, S16 Fig).

## Discussion

How do people’s error trends depend on the inference strategies they use? We examined the properties of errors made by human subjects performing a two-alternative forced-choice task with asymmetric evidence [7, 16, 17]. The evidence took the form of two colors of balls drawn from jars, such that one (“rare”) color was drawn less often than the other. Similar to ideal observers, most subjects exhibited a choice asymmetry favoring the option that produced fewer rare balls. In addition, subjects fell into two categories depending on the type of strategy that best described their responses. Subjects described by heuristic strategies, which were based on less information and fewer algorithmic operations, displayed substantially more choice variability but comparable choice asymmetry to the ideal observer. In contrast, subjects described by more-complex, mistuned Bayesian strategies displayed minimal increases in choice variability but much more bias than the ideal observer. These effects reflected the nature of the suboptimalities introduced by each strategy type: the heuristic strategies we considered did not take into account specific task features responsible for choice asymmetries and thus tended to add variability, whereas the Bayesian-like strategies that we considered did attempt to model those features explicitly but, when implemented suboptimally (mistuned) by the subjects, tended to exacerbate asymmetries inherent in such decision rules.

### Inversion of the bias-variance trade-off

These findings provide new insights into the generalizability of bias-variance trade-offs that are well established in machine learning and related fields [2, 3] and can be used to account for individual differences in human behavior under certain conditions [1, 4]. Bias-variance trade-offs can be conceptualized in terms of fitting various functions that differ in complexity (e.g., polynomial order) to noisy data whose generative source is unknown. Typically, simpler (e.g., linear) models tend to have higher bias, because they miss higher-order (e.g., nonlinear) features of the generative source, but lower variance, because their best-fitting parameters are relatively stable across different data instances. In contrast, more complex (e.g., high-order polynomial) models tend to have lower bias, because they can capture complex features of the data, but higher variance, because the specific features they capture can differ across different data instances.

Critically, this traditional conceptualization is based on the assumption that each model, regardless of its complexity, is “optimal,” using the best-fitting parameters given the data and thus does not introduce additional suboptimalities and errors. In contrast, we considered cases in which the proposed models (inference strategies) could differ in both complexity and (sub)optimality. Specifically, we considered two broad classes of strategies that could result in suboptimalities either from the model used or a mistuning of the parameters. In the context of asymmetric evidence, these suboptimalities introduced errors that could invert the bias-variance trade-off. However, this inversion only manifested when considering the relationship of complexity across model classes in asymmetric contexts. In contrast, decreases in complexity within a model class in asymmetric contexts produced increases in both bias and variance, regardless of model class. Therefore, our results suggest that the inversion of the bias-variance trade-off arises in particular situations, such as when suboptimal strategies are used in asymmetric environments, and may produce a potentially interesting way to analyze performance/complexity trends in models and subject data in future studies of human inference.

### Impacts of mistuned Bayesian strategies on the bias-variance trade-off

One notable component of the bias-variance inversion we observed in subjects’ responses is an exacerbation in choice asymmetry for Mistuned Bayesian strategies. In general, mistuning of Bayesian model parameters is not surprising, given that Bayesian models are computationally expensive [18] and difficult to tune appropriately [6, 19]. However, the nature of this mistuning for tasks involving asymmetric evidence is different than for more commonly studied tasks involving symmetric evidence, in several ways. These differences highlight specific challenges that an effective inference strategy must overcome and can be used to predict potential patterns in people’s response errors in asymmetric conditions.

First, a major factor governing performance on inference tasks with either symmetric or asymmetric evidence is the amount and/or quality of available observations. In general, inferences based on less evidence tend to be less accurate [20, 21], and the ideal observer does not show systematic biases to a particular alternative when the evidence and priors are symmetric (although such biases can arise from near-Bayesian decision strategies [22, 23]). In contrast, when evidence is limited and asymmetric, systematic choice asymmetries can be expected even for an ideal observer. As we have shown, people have a very strong tendency to exacerbate these asymmetries, even when given explicit instructions that the alternatives are equally likely. Thus, systematic biases might be a general feature of inferences that must operate on limited asymmetric evidence.

Second, effective inference requires weighting evidence appropriately. For symmetric conditions, this weighting should be calibrated to optimize choices but in general can be effective as long as the symmetry in the evidence weights is maintained, even if the evidence is mis-scaled relative to the true LLR [24]. In contrast, for asymmetric conditions this weighting often requires much more fine tuning that, when implemented suboptimally, can give rise to systematic errors. In our study, many subjects underweighted evidence from rare balls, which may reflect a bias toward evenly weighting the evidence gleaned by each ball type. Thus, a strong prior over even ball-weighting may pull subjects away from the ideal (asymmetric) weights. Moreover, the description-experience gap theory distinguishes the tendency to overestimate the importance of rare events when their frequency is described and underestimate their importance when subjects learn their frequency through experience [25–28]. For our tasks, event probabilities were both described and experienced across trials, which previously has been shown to promote better decisions [29]. Nevertheless, a substantial fraction of our subjects underweighted evidence from rare balls. Future iterations could compare this combined structure with one where subjects only experience the statistics of the jars to identify how universal this preference for underweighting evidence is in humans.

Third, many subjects used strategies that appeared to be based on subjective priors with a preference for the low jar. These findings are distinct from previous work that examined choice biases in tasks with symmetric evidence but asymmetries in expected choice frequencies [30–33] or reward outcomes [32, 34–37]. Under those conditions, biases based on asymmetric priors are common and, on average, tend to follow established, normative principles often formulated in the context of Signal Detection Theory [30] and/or sequential analysis [38]. In our study, subjects tended to either use inappropriate priors (e.g., subjects whose choices were best matched by the Prior Bayesian model with a prior biased towards the low jar) or neglect the symmetric prior altogether (e.g., subjects whose choices were best matched by heuristic models). These strategies could, in principle, reflect a relatively common form of recency bias that can cause an initial belief shift in the direction of the previous response [31, 32, 34, 35, 39, 40], and, more generally, is consistent with many previous findings of mistuned priors [41–45]. Alternatively, while our Prior Bayesian model described changes in choice asymmetry that were attributed to biased priors without impacts to the ideal evidence weights, it is plausible that the ideal observer model and its mistuned Bayesian variants could be implemented by a competitive neural network model with plastic synapses that could represent the evidence asymmetry of rare balls and asymmetric priors indicative of base rate neglect [46, 47].

### LLR-independent impacts on the bias-variance trade-off

Another important component of the inverted bias-variance trade-off was the relatively high variance for subjects who used heuristic versus Bayesian-like strategies. In the classic bias-variance trade-off, it is critical to distinguish variance (variability driven by sensitivity to noisy observations), which is anti-correlated with bias, from noise (variability driven by intrinsic factors), which is not generally predicted to relate to bias. Likewise, we attempted to distinguish the two sources of choice variability in terms of: 1) the mean absolute error of a subject’s choices, which we interpreted primarily as variance because it represents observation-specific (and LLR-independent) choice variability; and 2) the slope of the fit psychometric function, which we interpreted primarily as noise because it represents a general, LLR-dependent degradation of choice accuracy. Although both measures reflect both sources of variability to some extent, as evidenced by the correlations between the two, either metric was consistent with our interpretation, with heuristic models showing higher values of noise and variance.

Specifically, the Bayesian models added noise to an LLR-based decision variable, which affected the steepness of the (biased) psychometric function but less so observation-specific variability. In contrast, the heuristic models made probabilistic choices in an observation-dependent manner, which affected both the steepness of the psychometric function and the observation-specific variability. These results imply that, like for the classic bias-variance trade-off, the inverted form that we found is not just an empirical observation. Rather, it is an inherent information processing trade-off that depends on whether the suboptimal strategy operates primarily on latent (as in Bayesian-like strategies; e.g., LLR) or directly observable (as in heuristic strategies; e.g., rare ball count regardless of common ball count) properties of asymmetric environments.

### Causes of suboptimal behavior

Why do people typically behave suboptimally in cognitive tasks? Subjects have diverse individual definitions of optimality, which can be different from the task goals [19]. Likewise, suboptimal behavior may be a result of computational and cognitive limits of the brain, which may hinder a subject’s ability to optimally tune or perform complex tasks [12, 18, 48]. Attention also varies across subjects, and attention levels may correlate with the likelihood of using a Bayesian or heuristic strategy and modulate the amount of mutual information between observations and their responses [49]. Moreover, the presence and amplitude of rewards shapes task attention [50], which could be reflected in strategy usage.

In this task, suboptimality took three forms: 1) underweighting rare balls; 2) biased priors in favor of the low jar; and 3) applying heuristics, which occurred predominantly in harder tasks. We hypothesize that underweighting may be the result of weighting biases in favor of symmetric weights, rather than a mistuning relative to the ideal-observers weights, given that subject’s rare-ball parameters showed comparable values for both easy and hard asymmetric blocks. Likewise, the mistuning of subjects’ priors in favor of the low jar may reflect a recency bias, in which previous low-jar responses encourage subjects to repeat their choice [51, 52]. Finally, the use of heuristic strategies in more complex tasks (e.g., hard asymmetric block where inference is more difficult) can often approximate the accuracy of a more complex model [5]. Whereas heuristics fail to perform as well in this task, it is possible that subjects have previously learned that such shortcuts are beneficial by reducing computational cost without forfeiting accuracy. Given that subjects were not provided feedback on their responses, it is reasonable for them to apply previous experience to this task. Such possibilities account only partly for the diversity of causes which lead people to perform suboptimal inference in our task, but future work could explore how different rewards affect strategy form, complexity, and optimality.

## Conclusion

By studying human inferences based on observations of asymmetrically available evidence, we identified a novel inversion of the classic bias-variance trade-off that arises as a result of the strong tendency of people to mistune Bayesian strategies further along the direction of existing choice asymmetries. This finding also demonstrates the power of de-tuning Bayesian models as a way of distinguishing strategies in a human cohort. Our study of strategy complexity also distinguished Bayesian-like and heuristic models based on the mutual information between observations and responses, in addition to their distinct choice error trends. In general, probing how humans make inferences in the presence of asymmetric evidence highlights relationships between bias, variance, complexity, and human error that cannot be observed in standard decision tasks and provides unique insight into the basis of human idiosyncrasies and bias-variance trade-offs for suboptimal inference strategies.

## Supporting information

S1 Text Task and Recruitment. (DOCX)

S2 Text Model fitting. (DOCX)

S3 Text Subject model fitting. (DOCX)

S4 Text Choice-Asymmetry Analyses. (DOCX)

S5 Text Noise Versus Variance. (DOCX)

S6 Text Symmetric results. (DOCX)

S7 Text Complexity analyses. (DOCX)

S1 Fig Example of the screen viewed by subjects on Amazon Mechanical Turk. The details of the current set of jars were available to participants on every trial. A prompt at the bottom of the screen indicated to the subject to select the jar from which the sample was drawn.
(TIF)

S2 Fig Inattentive subjects. Accuracy for each subjects’ interspersed control trials to test for attentiveness (3 interspersed blocks of 12 trials). Inattentive subjects were defined as those whose accuracy was 50% or lower on two or more interspersed control blocks (3 subjects identified, red lines). These subjects were excluded from all further analyses.
(TIF)

S3 Fig Trial identification. Examples of the Bayesian parametric posteriors of the Noisy Bayesian model with a flat prior over the noise variance 0 ≤ *a* ≤ 1 and the rare-ball weight 0 < *ρ* ≤ 24.16 (computed from jars with rare-ball probabilities 0.01 ≤ *h*_±_ ≤ 1). Posteriors are based on synthetic responses from a Noisy Bayesian model whose true parameters use the ideal observer’s *ρ* and a low level of noise (*a* = 0.1) and are collected for varied block lengths (12, 24, and 60 trials, columns) of the Hard Asymmetric (HA) and Easy Asymmetric (EA) blocks (rows). True parameters used to generate responses are shown as blue dots. By 60 trials, the parameters are well identified in the posterior, with >40% of the posterior falling within a one parameter-value range of the true parameter (green box, corresponding percentages shown in green on top of each panel). Because a flat prior is used, there is a high likelihood for alternative scenarios in which there is a trade-off between higher noise and lower *ρ* values, as shown by the arrows in the HA fits and motivated the use of an informative prior for Bayesian model parameter recovery (see Methods and S4 Fig).
(TIF)

S4 FigInformed priors.The weakly informative prior used for Bayesian model fitting, computed from the pilot data of 20 subjects. Posteriors were computed for each subject based on the Noisy Bayesian model with a flat prior and then averaged to produce a population posterior for each block. The averaged posterior was then smoothed to create an informative prior used during subsequent model fitting. To smooth the posterior with respect to *ρ*, the averaged marginal posterior was filtered using a normal distribution $N(\mu ,\sigma^{2})$, where the mean *μ* was set at the maximum value of the averaged marginal posterior and the variance *σ*^2^ was set such that the median mean squared error (MSE) of the parameter fits *ρ* for 100 synthetic Noisy Bayesian datasets was below one. The averaged marginal posterior with respect to the noise parameter *a* was smoothed using the function (*x* + *c*)/(1 + *cL*), where *x* is the marginal posterior and *c* and *L* are scaling constants selected such that the averaged posterior was smooth (no jagged edges) but did not impact the accuracy of the rare-ball parameter fitting (symmetric blocks: L = 2, C = 5, asymmetric blocks: L = 1, C = 2). Red line shows the rare-ball weighting *ρ* for the ideal observer in each block.)(TIF)

S5 Fig Model identification. Fraction of times an alternative model was correctly identified as compared to the Noisy Bayesian model using Bayes factors for each block: Control (CT), Hard Asymmetric (HA), Hard Symmetric (HS), Easy Asymmetric (EA), Easy Symmetric (ES). 100 sets of synthetic responses were produced for every model using the human task structure (4 blocks with 42 trials, control block with 60 trials). The Noisy Bayesian model includes noise and a rare-ball weight, *ρ*, that varies across subjects. The Noisy Bayesian Set *ρ* model (set *ρ*) assumes that *ρ* equals the ideal observer’s rare-ball weight (*ρ*_*IO*_). The Prior Bayesian model (Prior) includes a jar bias (prior), and assumes *ρ* = *ρ*_*IO*_. The Asymmetric (Asym) model assumes an asymmetric repetition bias following a low-jar response. The Prior with Variable *ρ* (Prior var *ρ*) model is the noisy Bayesian model with biased prior. The Windowing (Wind) model assumes a set window of evidence for each trial. The Variable Rare Ball (Var Rare) model sets the probability of response for the high jar based on whether or not the number of observed rare balls meets some threshold. The Rare Ball model (Rare) is a reduction of the Variable Rare Ball model and sets the rare ball threshold to 1 (observing any rare ball corresponds with a high jar response of probability *P*_*rare*_). The History Dependent Rare Ball (HD Rare) model incorporates past trial responses into the Rare Ball model. Under the Guess model (Guess), the high jar is chosen with some probability that is set as a free parameter, regardless of the balls observed. Models were included in subject analyses only if synthetic responses were identifiable above 80% for all blocks (Asym and Prior var *ρ* excluded) and if > 5 subjects were best fit by the model in any given block (Wind and HD rare models were excluded from further analyses).
(TIF)

S6 Fig Consistent subject model fits. Fraction of subjects who were best fit by models in the same class, Bayesian (purple) or Heuristic (yellow). Subjects’ best-fit strategies were compared across all blocks (All), only asymmetric blocks (Asym) or only symmetric blocks (Sym). Subjects were typically best described by different models within the model class for each block.
(TIF)

S7 Fig Subject model accuracy. Subject accuracy based on each subject’s best-fit model in a block: Control (CT), Hard Asymmetric (HA), Hard Symmetric (HS), Easy Asymmetric (EA), Easy Symmetric (ES). Colored dots represent individual subject accuracy. Black diamonds and errorbars show the bootstrapped means (1000 iterations) and 95% confidence interval for each model-block. Accuracy was significantly (*p* < 0.05) above chance (0.5) for all models.
(TIF)

S8 Fig Subject cross validation. 10-fold 90/10 cross-validation accuracy performed between each subject and the model that best describes their responses for each block: Control (CT), Hard Asymmetric (HA), Hard Symmetric (HS), Easy Asymmetric (EA), Easy Symmetric (ES). Each colored point represents one individual. Black diamonds and errorbars show the bootstrapped means (1000 iterations) and 95% confidence interval for each model-block. Cross-validation accuracy was significantly above chance (0.5; *p* < 0.05) for all models except the Rare-Ball model in the HS block and the Guess model in all blocks. Mean cross-validation accuracy was ≥ 0.8 for all models except the Rare-Ball and Guess model. Ranges (across blocks) for the percentage of subjects with ≥ 80% cross validation accuracy for each model: Noisy Bayesian (Noisy): 40-100%; Noisy Bayesian Set *ρ* (set *ρ*): 54-85%; Prior Bayesian (Prior): 76-100%; Variable Rare Ball (Var): 50-100%; Rare Ball (Rare): 0-38%; Guess: 0-22%.
(TIF)

S9 Fig Choice asymmetry. Left: Low-jar response fractions as sample lengths (number of balls observed) changes for subjects and sample-matched ideal observer (model) for asymmetric blocks (Hard Asymmetric (HA), Easy Asymmetric (EA)). Bold markers and errorbars are bootstrapped means and 95% confidence intervals. Filled markers denote a significant population shift away from 0.5 (*p* < 0.05). Center: For the asymmetric blocks, the ideal observer’s probability of responding correctly in favor of the low or high jar changes with the number of balls drawn and the jar asymmetries (*h*_±_). As the likelihood of observing a rare ball increases, the probability of choosing the low jar decreases, until reaching a discrete shift in the number of rare balls that must be drawn (e.g., 1 up to 2) to trigger a “high” response, generating a sawtooth-shaped response fraction function of ball number. Right: The overall (correct and incorrect trials) low-jar response probability for the ideal observer shows a general decrease in choice asymmetry as sample size increases. However, the effect is accompanied by the sawtooth structure depicted in the center panels.
(TIF)

S10 Fig Noise variance comparison. Top: Estimated noise and variance from psychometric functions fit to individual subject data (points). Noise and variance showed a significant correlation in all blocks: Control (CT), Hard Asymmetric (HA), Hard Symmetric (HS), Easy Asymmetric (EA), Easy Symmetric (ES) (Spearman’s Correlation, *p* < 0.05). Center: Same data as in the top row, but color coded by each subject’s best-fit models for each block. In general, heuristic subjects had the largest values of variance and noise. Triangles represent medians for each model group. Filled triangles differ significantly from the Nearly Ideal subjects (two-sided Wilcoxon rank-sum test, *p* < 0.05). Bottom: Noise and variance values from synthetic responses generated by each subject’s best-fit model and parameters (198 sets of synthetic responses distributed across models based on the subject strategies from Fig 4D). Both subject and synthetic data showed similar relationships between noise and variance, with Bayesian models displaying less noise and variance than heuristics. For all plots, large noise values (>20) were rescaled to 20 for visualization purposes.
(TIF)

S11 Fig Noise bias comparison. Subjects’ estimated bias and noise based on the best-fit psychometric functions shown for each task block: Control (CT), Hard Asymmetric (HA), Hard Symmetric (HS), Easy Asymmetric (EA), Easy Symmetric (ES). Here, dots represent individual subjects, color coded by an individual’s best-fit strategy. Triangles represent medians for each model group: the Nearly Ideal subjects, Mistuned Bayesian subjects, and Heuristic subjects. Filled triangles significantly differed from the Nearly Ideal subjects based on a two-sided Wilcoxon rank-sum test with *p* < 0.05. Large noise values (> 20) were rescaled to 20 for visualization purposes. Results mimicked those observed when using our measure of variance (see main text) instead of noise.
(TIF)

S12 Fig Symmetric block results. Subject bias and variance on symmetric blocks: Control (CT), Hard Symmetric (HS), and Easy Symmetric (ES), as in Figs 3C–3F and 5. Top: Median high-jar responses (points) and best-fitting logistic psychometric functions. Bottom: Bias and variance based on the best-fit psychometric function. Points reflect individual subjects, color-coded by subjects’ best-fit models. Triangles represent medians for each model group: the Nearly Ideal subjects, Mistuned Bayesian subjects, and Heuristic subjects. Filled triangles significantly differed from the Nearly Ideal subjects based on a two-sided Wilcoxon rank-sum test with *p* < 0.05.
(TIF)

S13 Fig Complexity correlations. Bias and variance tended to decrease with complexity (MI) across subjects grouped by strategy, particularly on asymmetric blocks. Top: bias-MI plots as in Fig 6C for all blocks (columns, as indicated). Points are data from individual subjects, color coded by their best-fit strategy. Significant correlations (Spearman correlation, *p* < 0.05) are shown for each model group using color-coded lines. Only asymmetric blocks showed significant (negative) correlations, implying that within groups, bias tended to increase with decreasing strategic complexity. Bottom: variance-MI plots as in Fig 6D for all blocks, plotted as in the top row. All blocks showed at least one within-group relationship between complexity and variance, consistent with general trends of better (less variable) performance associated with more-complex strategies.
(TIF)

S14 Fig Mutual information with previous response. Across-group bias-variance relationships were robust to a measure of mutual information (MI) that took into account not just the balls observed on the current trial (i.e., relevant information, as in Fig 6A)) but also the previous choice (i.e., irrelevant information), for the two asymmetric blocks (columns, as indicated). **a**: Accuracy versus MI. The bound is the maximum accuracy attainable by the idea observer for a fixed MI in the limit of many trials. Note that points could exceed the asymptotic accuracy bound because the number of trials for each subject was finite. The dashed horizontal lines indicate the accuracy bound for maximum MI values. X’s are data from individual subjects. Squares are per-group medians (filled symbols for Mistuned Bayesian and Heuristic groups indicate that the median MI is significantly different from that of the Nearly Ideal group median, Wilcoxon rank-sum test, *p* < 0.05). Including past choices tended to give slightly higher MI measures but maintain the same ordering from Heuristics (simplest), to Mistuned Bayesian, to Nearly Ideal (most complex; compare to Fig 6A)).**b**: Difference in MI using this measure versus MI without the previous choice. X’s are data from individual subjects. Squares are per-group medians (filled symbols for Mistuned Bayesian and Heuristic groups indicate that the ordinate value is significantly different from that of the Nearly Ideal group median, Wilcoxon rank-sum test, *p* < 0.05). In general, including the previous choice increased MI (i.e., subjects tended to have sequential choice dependencies) but did not affect the inverted bias-variance trade-off.**c**: Bias-MI and variance-MI plots using this MI measure that includes the previous choice.
(TIF)

S15 Fig Simulated response complexity. Synthetic sets of responses were produced using each subject’s best-fit model and parameters and new samples of ball draws (198 sets of synthetic responses distributed across models based on the strategies that best describe subjects’ responses from Fig 4D) for each block: Control (CT), Hard Asymmetric (HA), Hard Symmetric (HS), Easy Asymmetric (EA), Easy Symmetric (ES). Synthetic responses were then fit to psychometric functions with bias and variance values extracted. Each dataset of synthetic responses is denoted by a colored point associated with the generating model. Triangles show medians for each group. In asymmetric blocks, Mistuned Bayesian models show bias.
(TIF)

S16 Fig Algorithmic complexity. Algorithmic complexity [9] for each model was computed based on the number of operations performed on a trial, broken into: arithmetic, writing to memory, reading from memory, and storage operations. Heuristic models have lower complexity (yellow) compared to Bayesian models (purple). Bayesian model complexity varies with the number of balls observed (*n*). Example computations are shown for sample lengths of 2,5, and 10 balls. Computations were based on the following operations involved in each strategy:
**Guess:** Read and store parameter *P*_*guess*_.**Rare Ball:** Identify presence of the rare ball (max), read the probability of response, store *P*_*rare*_ and *P*_*no*_.**Variable Rare Ball:** All elements from the Rare-Ball model with additional operations to compute the number of rare balls and store the rare-ball threshold *θ*.**Noisy Bayesian Set *ρ*:** Multiplication of the ball weight for each ball observed (*n*) and *n* − 1 summations.**Noisy Bayesian:** Arithmetic as in the Noisy Bayesian Set *ρ* model with additional operations to read and store the rare-ball weight *ρ*.**Prior Bayesian** Arithmetic as in the Noisy Bayesian model with inclusion of the prior that is read and stored.
(TIF)
