# Supplementary material for: Suboptimal human inference can invert the bias-variance trade-off for decisions with asymmetric evidence
Source: PLoS Comput Biol. 2022 Jul 19;18(7):e1010323. doi: 10.1371/journal.pcbi.1010323 (PMC9337699; doi:10.1371/journal.pcbi.1010323)
Supplement: S1 Text — (DOCX) [file pcbi.1010323.s001.docx]

**Task and Recruitment**

The jar-discrimination task was performed using Amazon Mechanical Turk and a preregistered task structure doi:10.17605/OSF.IO/J9XET, which defined the number of trials, block types, display details, and sample lengths. The task details were determined by synthetic model fitting and comparisons to identify the task parameters that would allow for model identifiability when fitting subject data, while retaining a reasonable time length for testing. An example of the task depiction used while testing subjects is shown in S1 Fig.

To ensure attentive participation, we restricted subject recruitment (see Methods for inclusion and exclusion criteria) and included a training block with 24 control trials to identify attentive participation. Subjects who scored <80\%$accuracy on the training block were not allowed to complete the rest of the testing blocks and were not counted in our subject numbers (201 subjects total). Additionally, we motivated continued participation by providing payment to all subjects who completed all testing blocks. We also included a post-hoc attention analysis, which identified subjects who may have completed the testing blocks but stopped actively participating in this task. Specifically, we incorporated three short, 12-trial control blocks between each testing block. Subjects who achieved 50\% or lower on at least 2/3 control blocks were considered inattentive and not included in further analyses (3/201 subjects; S2 Fig)
