# Supplementary material for: Suboptimal human inference can invert the bias-variance trade-off for decisions with asymmetric evidence
Source: PLoS Comput Biol. 2022 Jul 19;18(7):e1010323. doi: 10.1371/journal.pcbi.1010323 (PMC9337699; doi:10.1371/journal.pcbi.1010323)
Supplement: S2 Text — (DOCX) [file pcbi.1010323.s002.docx]

**Model Fitting**

Our selection of the number of trials in a block was based on synthetic data produced using our baseline Noisy Bayesian model. We simulated responses from the Noisy Bayesian model with parameters selected randomly from the distributions for the rare-ball weight $\rho$ (computed for varying values of $h_{\pm}$ between 0.01–1) and noise (ranging between 0–1). We then performed parametric model fitting by computing the joint posterior of the parameters using the responses over x trials, where the number of trials x was varied. As shown in S3 Fig, the posteriors were broad and not well localized when the number of trials was low (<30) but had much smaller variance and means close to the true parameters for >40 trials. However, when the value of the noise parameter was high, fitting the Noisy Bayesian model to data resulted in lower values of $\rho$ (underweighting the rare ball). This trade-off was particularly noticeable when fitting to data obtained in asymmetric environments.

To mitigate concerns of misfitting resulting from this trade-off, we used an informative prior when model fitting across all blocks. We constructed this informative prior by fitting the Noisy Bayesian model to pilot data from 20 human subjects. The joint parametric posteriors were averaged across subjects to provide a preliminary estimate of the relevant parameters. Notably, because typical model fits to subject data indicated that noise was low, concerns about underweighting $\rho$ , the rare-ball weight, were reduced. For this reason, we used a smoothed version of the averaged posteriors as an informative prior for all subsequent model fitting with human data. Details about this smoothing process and the informative priors can be found in S4 Fig.

We next checked the identifiability of our models using Bayes factors and synthetically generated data for each alternative model. For each model, we generated 100 simulated data sets selecting parameters from the model distributions. For Bayesian models, model parameters were selected randomly based on the prior, which was peaked at low noise. Heuristic model parameters were selected from uniform distributions describing the probability of a particular response. We then fit the Noisy Bayesian and the true (simulated) model to each data set and computed the corresponding Bayes factors. If the $\log\left( BayesFactor \right)$ was a positive value, the true model was correctly identified. The fraction of correctly identified datasets for each model-task block is shown in S5 Fig. Only models that were correctly discriminated from the Noisy Bayesian model >80% of the time were included in our subject model fitting. Models that were later selected <5 times per block for human subjects were also removed from subsequent analyses.
