# Supplementary material for: Suboptimal human inference can invert the bias-variance trade-off for decisions with asymmetric evidence
Source: PLoS Comput Biol. 2022 Jul 19;18(7):e1010323. doi: 10.1371/journal.pcbi.1010323 (PMC9337699; doi:10.1371/journal.pcbi.1010323)
Supplement: S3 Text — (DOCX) [file pcbi.1010323.s003.docx]

**Subject Model Fitting**

Each subject performed multiple symmetric (ES and HS) and asymmetric (EA and HA) blocks. We tested whether the idiosyncratic, strategy-dependent relationships between choice bias and variance evident within blocks reflected the consistent or inconsistent use of particular strategies by individual subjects across blocks. We found that each subject's choice data were not typically best fit by the same model across blocks. However, there was more consistency in their use of classes of strategy (Bayesian or heuristic), particularly in terms of a relatively strong tendency for subjects to use Bayesian strategies across blocks (S6 Fig)

We also compared subject accuracy in each block with respect to the best-fit model to ensure that subject responses and model fits were reasonable. Accuracy for subjects best described by Bayesian models and by the heuristic Variable Rare Ball and Rare Ball models were significantly above chance levels for all blocks (S7 Fig, bootstrapped means and 95% confidence intervals, p<0.05). Subjects identified as using the Guess model had lower accuracy, as expected.

We confirmed the model selections using 10-fold 90/10 cross-validation. Overall, all the selected models except for the Guess model (which does not predict trial-to-trial responses) provided good predictions of the subjects' trial-to-trial responses. The responses of subjects identified as using Bayesian strategies were predicted with >80% accuracy, on average. Heuristic Variable Rare Ball and Rare Ball models also predicted subject responses significantly above chance levels and outperformed the Guess model (S8 Fig; bootstrapped means and 95% confidence intervals p<0.05).
