# Supplementary material for: Suboptimal human inference can invert the bias-variance trade-off for decisions with asymmetric evidence
Source: PLoS Comput Biol. 2022 Jul 19;18(7):e1010323. doi: 10.1371/journal.pcbi.1010323 (PMC9337699; doi:10.1371/journal.pcbi.1010323)
Supplement: S4 Text — (DOCX) [file pcbi.1010323.s004.docx]

**Choice-Asymmetry Analyses**

In addition to overall choice asymmetry, we considered the effect that sample length had on responses in favor of the low jar. In general, the ideal observer's choice asymmetry is reduced as the sample length increased or the task was made easier (EA had less asymmetry than HA, S9 Fig. However, this trend is nonmonotonic because of abrupt jumps in the threshold of rare balls needed to trigger a high-jar response (high-jar response threshold $B$), which changes with the sample length $n$. Specifically, when considering the probability of a correct response (e.g., respond low jar when it is low jar), the ideal observer showed a trade-off in response probability for each jar based on $B$, which is dependent on $h_{+}$ and $h_{-}$. For symmetric cases ($h_{+}=1-h_{-}$), $B=n/2$. For asymmetric cases, $B<n/2$if $h_{+}+h_{-}<1$Therefore, the sample lengths where $B$changes caused a flip in jar probabilities. Overall, low-jar biases tended to decrease with increased sample size. However, for small changes in the ball count, there could be abrupt changes in the low-jar response fraction, when the high-jar response threshold crossed an integer value. In contrast to the ideal observer, subjects showed a gradual decrease in asymmetry as a population with no abrupt changes.
