# Supplementary material for: Suboptimal human inference can invert the bias-variance trade-off for decisions with asymmetric evidence
Source: PLoS Comput Biol. 2022 Jul 19;18(7):e1010323. doi: 10.1371/journal.pcbi.1010323 (PMC9337699; doi:10.1371/journal.pcbi.1010323)
Supplement: S5 Text — (DOCX) [file pcbi.1010323.s005.docx]

**Noise Versus Variance**

We decomposed choice variability into two components defined with respect to the logistic psychometric functions: 1) noise, which we defined as the steepness of the function such that shallower functions correspond to higher noise; and 2) variance, which we defined as the mean absolute error between the data and the best-fitting psychometric function and represents choice patterns that were independent of the smooth, LLR-based function. Despite measuring different features of the choice data, these two metrics were correlated with each other for individual subjects in each task block (S10 Fig). This correlation implied that the two metrics together reflected choice variability that is central to this study. We also found that subjects best fit to heuristics tended to have more variance and noise, as compared to the Bayesian subjects. This relationship between noise and variance was retained when synthetic data were used.

Using noise instead of variance produced the same deviations from the ideal observer in asymmetric blocks. Comparing noise and bias, we found that heuristic subjects showed larger noise values but little bias, whereas Mistuned Bayesian subjects showed bias with little noise (S11 Fig).
