# Supplementary material for: Suboptimal human inference can invert the bias-variance trade-off for decisions with asymmetric evidence
Source: PLoS Comput Biol. 2022 Jul 19;18(7):e1010323. doi: 10.1371/journal.pcbi.1010323 (PMC9337699; doi:10.1371/journal.pcbi.1010323)
Supplement: S6 Text — (DOCX) [file pcbi.1010323.s006.docx]

**Symmetric Results**

The subjects' choices were closer to the ideal observer's choice in symmetric blocks as compared to asymmetric blocks. Specifically, subjects showed almost no bias, and response deviations from the ideal observer were less frequent and almost always the result of increased variance (S12 Fig, top). Similar to asymmetric blocks, variance tended to be highest for subjects who were best fit by heuristic models (S12 Fig, bottom).
