# Supplementary material for: Suboptimal human inference can invert the bias-variance trade-off for decisions with asymmetric evidence
Source: PLoS Comput Biol. 2022 Jul 19;18(7):e1010323. doi: 10.1371/journal.pcbi.1010323 (PMC9337699; doi:10.1371/journal.pcbi.1010323)
Supplement: S7 Text — (DOCX) [file pcbi.1010323.s007.docx]

**Complexity Analyses**

We examined relationships between bias and variance with respect to two complementary measures of complexity, one based on the information bottleneck, and the other on algorithmic complexity (see text and Methods for more detailed descriptions of the two measures). Here we present several findings that support our main conclusions that subjects that used less-complex heuristic strategies (assessed via either complexity measure) tended to have relatively high variance but low bias, whereas subjects that used more-complex Bayesian-based strategies tended to have relatively low variance but high bias.

For the information-bottleneck measure, the bias-variance relationships were evident across subjects grouped by strategy type (i.e., heuristic versus Bayesian), despite the fact that there was an overall decrease in bias and variance with increased complexity within each group, especially on asymmetric blocks (S13 Fig). These relationships were robust to inclusion of the subject's choice from the previous trial (irrelevant information), in addition to the balls presented on the current trial, in the ``observation'' used to compute MI (S14 Fig).

To confirm that the observed complexity trade-off was not the result of idiosyncratic behaviors not well captured by the models, we simulated choice data using the best-fitting models (S15 Fig). Specifically, simulated choices were generated using the best-fitting model and parameters for each subject and block, applied to new ball samples. These simulated choices exhibited the same relationship of relatively high variance and low bias for the Heuristic models and relatively low variance and high bias for the Mistuned Bayesian models (S15 Fig).

We also considered algorithmic complexity [1], which has previously been considered in the context of limits and trade-offs in human inference [2-4]. Algorithmic complexity assigns computational costs to each component of the strategy by simply counting the total number of operations (arithmetic, writing to memory, reading from memory, and storage) needed to perform the task (detailed breakdown in S16 Fig). Based on our assignments, this metric showed a sample-length-dependent scaling in Bayesian complexity, but still confirmed that measures of complexity for the Bayesian models were much larger than that of heuristics, on average as shown in Fig 6E. While alternative reasonable costs could be assigned for each operation, these approaches would merely amplify our results.

References

1. Tavoni G, Balasubramanian V, Gold JI. What is optimal in optimal inference?
   Curr Op Behav Sci. 2019;29.
2. Bossaerts P, Murawski C. Computational Complexity and Human
   Decision-Making. Trends Cog Sci. 2017;21
3. Bossaerts P, Yadav N, Murawski C. Uncertainty and computational complexity.
   Phil Trans Roy Soc LondSeries B. 2019;374
4. Kool W, Gershman SJ, Cushman FA. Planning Complexity Registers as a Cost
   in Metacontrol. J Cog Neurosci. 2018;30
