# Supplementary figures and images for: Suboptimal human inference can invert the bias-variance trade-off for decisions with asymmetric evidence
Source: PLoS Comput Biol. 2022 Jul 19;18(7):e1010323. doi: 10.1371/journal.pcbi.1010323 (PMC9337699; doi:10.1371/journal.pcbi.1010323)

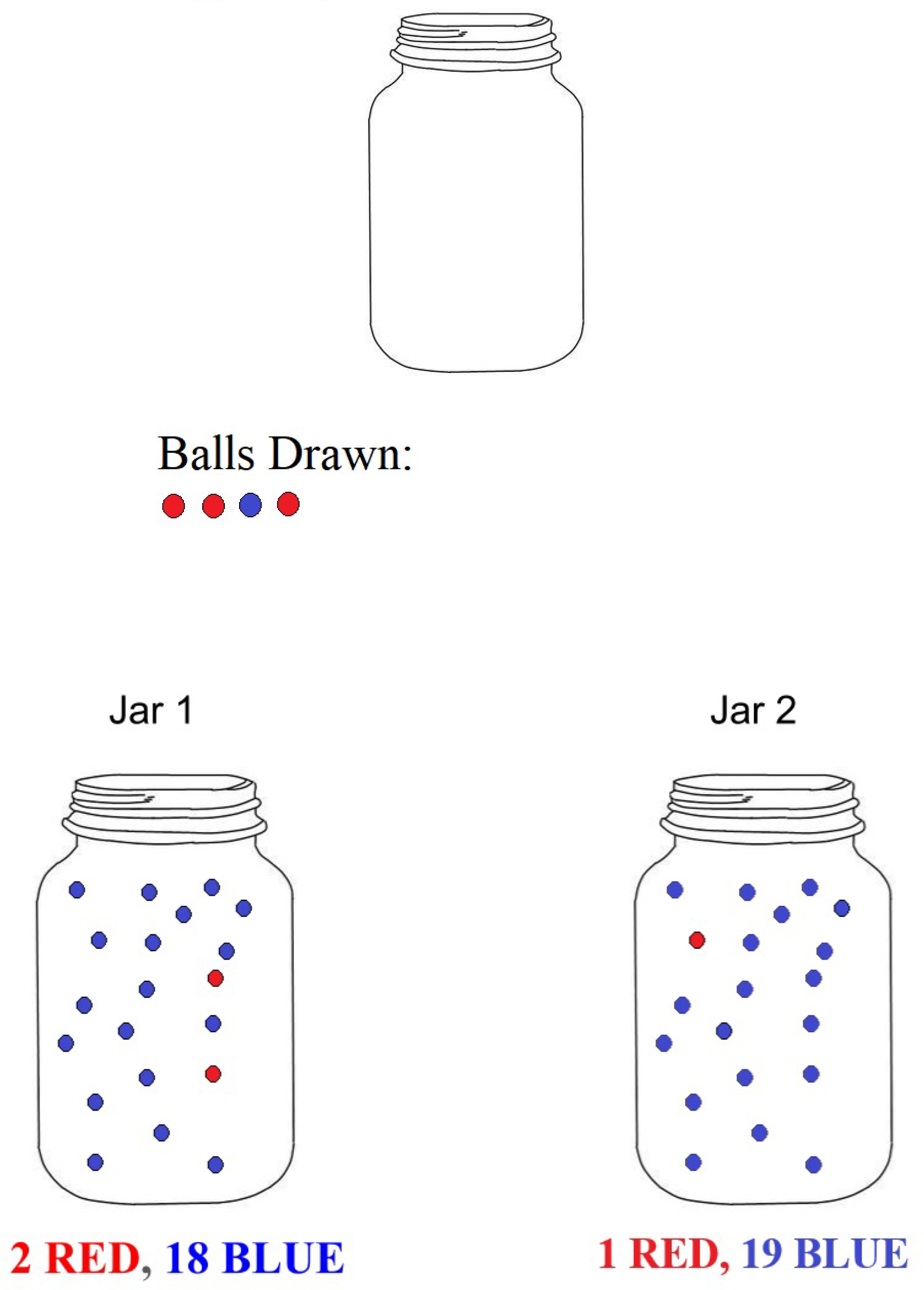

Supplement: S1 Fig — The details of the current set of jars were available to participants on every trial. A prompt at the bottom of the screen indicated to the subject to select the jar from which the sample was drawn. (TIF) [file pcbi.1010323.s008.tif]

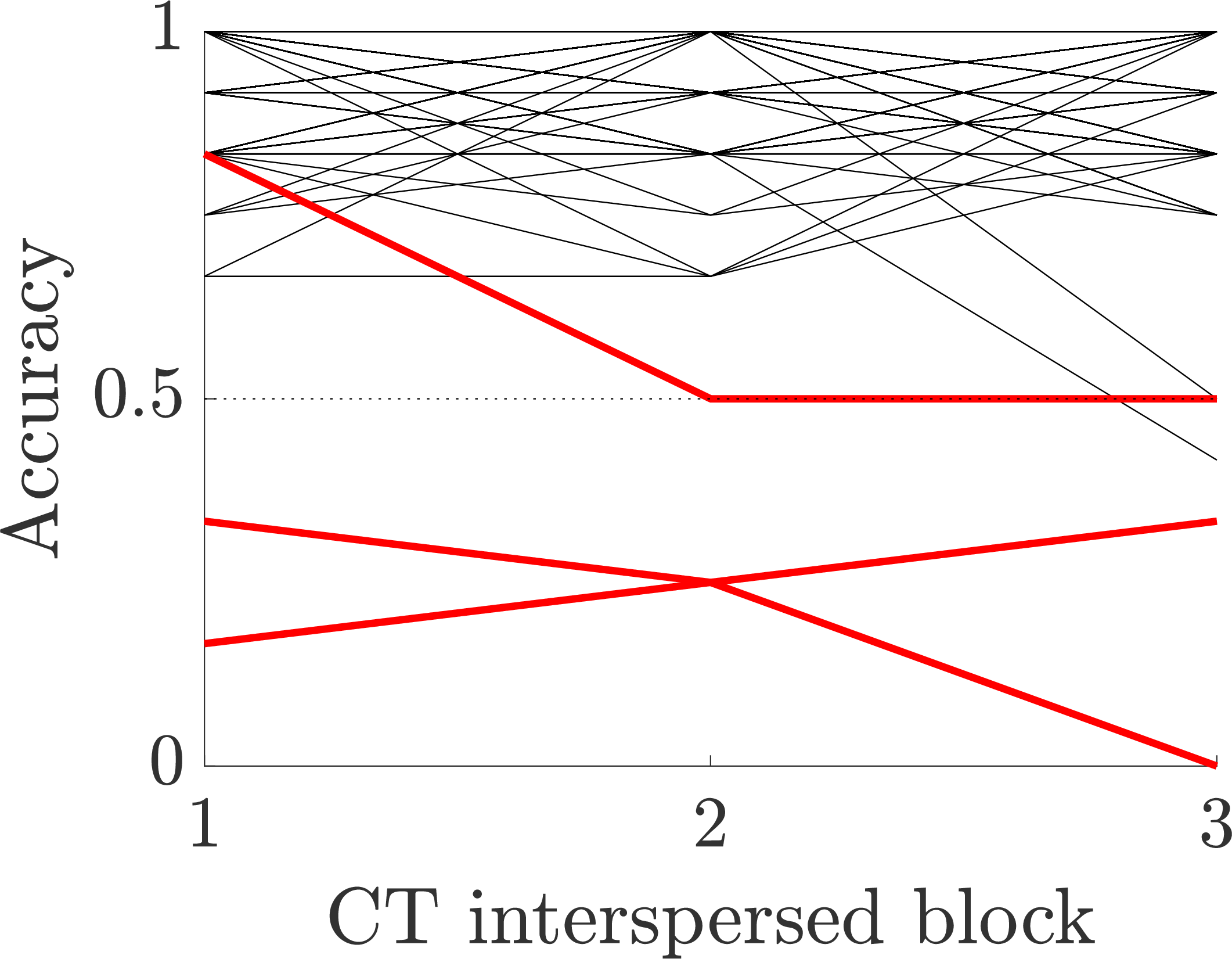

Supplement: S2 Fig — Accuracy for each subjects’ interspersed control trials to test for attentiveness (3 interspersed blocks of 12 trials). Inattentive subjects were defined as those whose accuracy was 50% or lower on two or more interspersed control blocks (3 subjects identified, red lines). These subjects were excluded from all further analyses. (TIF) [file pcbi.1010323.s009.tif]

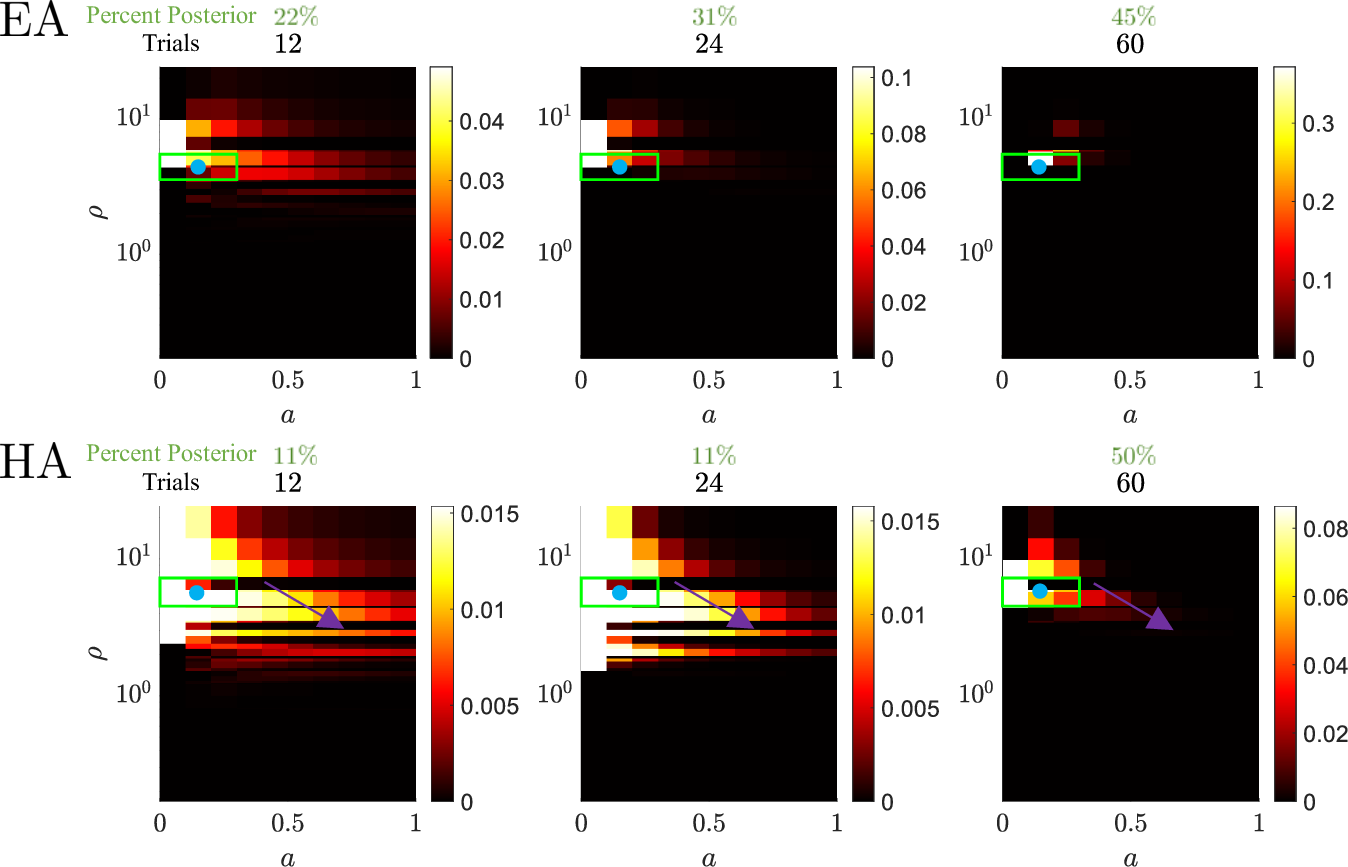

Supplement: S3 Fig — Examples of the Bayesian parametric posteriors of the Noisy Bayesian model with a flat prior over the noise variance 0 ≤ a ≤ 1 and the rare-ball weight 0 < ρ ≤ 24.16 (computed from jars with rare-ball probabilities 0.01 ≤ h± ≤ 1). Posteriors are based on synthetic responses from a Noisy Bayesian model whose true parameters use the ideal observer’s ρ and a low level of noise (a = 0.1) and are collected for varied block lengths (12, 24, and 60 trials, columns) of the Hard Asymmetric (HA) and Easy Asymmetric (EA) blocks (rows). True parameters used to generate responses are shown as blue dots. By 60 trials, the parameters are well identified in the posterior, with >40% of the posterior falling within a one parameter-value range of the true parameter (green box, corresponding percentages shown in green on top of each panel). Because a flat prior is used, there is a high likelihood for alternative scenarios in which there is a trade-off between higher noise and lower ρ values, as shown by the arrows in the HA fits and motivated the use of an informative prior for Bayesian model parameter recovery (see Methods and S4 Fig). (TIF) [file pcbi.1010323.s010.tif]

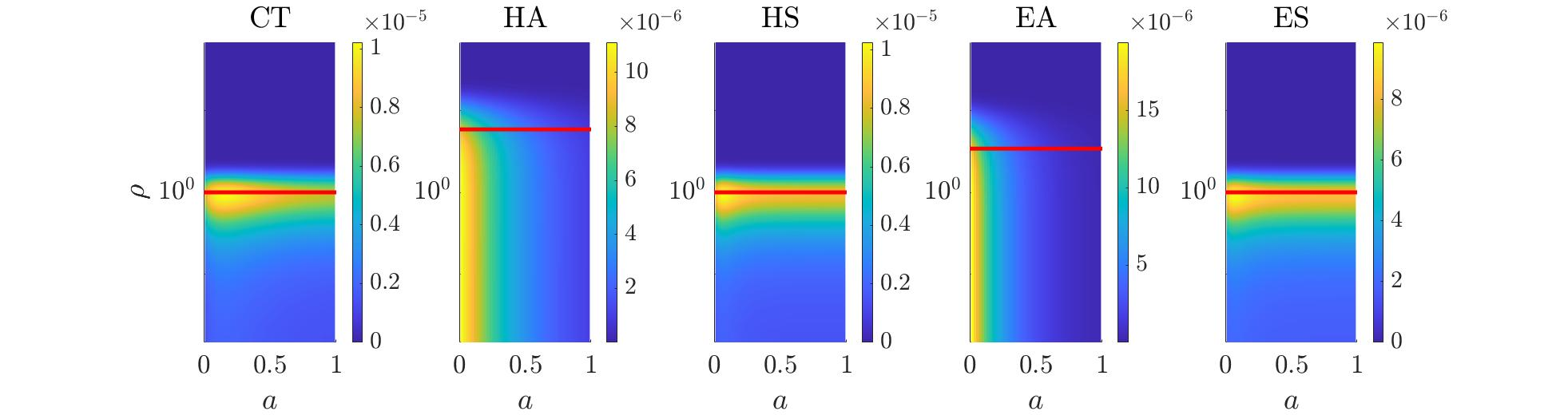

Supplement: S4 Fig — The weakly informative prior used for Bayesian model fitting, computed from the pilot data of 20 subjects. Posteriors were computed for each subject based on the Noisy Bayesian model with a flat prior and then averaged to produce a population posterior for each block. The averaged posterior was then smoothed to create an informative prior used during subsequent model fitting. To smooth the posterior with respect to ρ, the averaged marginal posterior was filtered using a normal distribution N(μ,σ2), where the mean μ was set at the maximum value of the averaged marginal posterior and the variance σ2 was set such that the median mean squared error (MSE) of the parameter fits ρ for 100 synthetic Noisy Bayesian datasets was below one. The averaged marginal posterior with respect to the noise parameter a was smoothed using the function (x + c)/(1 + cL), where x is the marginal posterior and c and L are scaling constants selected such that the averaged posterior was smooth (no jagged edges) but did not impact the accuracy of the rare-ball parameter fitting (symmetric blocks: L = 2, C = 5, asymmetric blocks: L = 1, C = 2). Red line shows the rare-ball weighting ρ for the ideal observer in each block.) (TIF) [file pcbi.1010323.s011.tif]

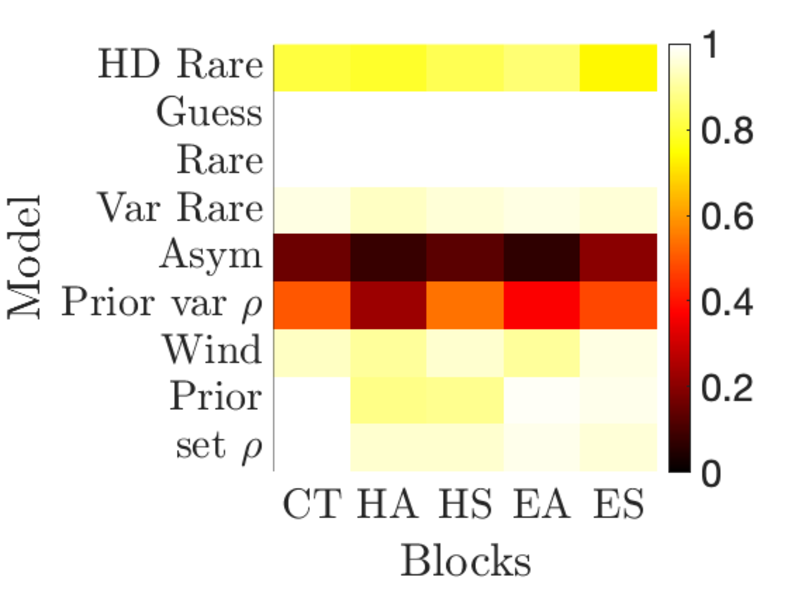

Supplement: S5 Fig — Fraction of times an alternative model was correctly identified as compared to the Noisy Bayesian model using Bayes factors for each block: Control (CT), Hard Asymmetric (HA), Hard Symmetric (HS), Easy Asymmetric (EA), Easy Symmetric (ES). 100 sets of synthetic responses were produced for every model using the human task structure (4 blocks with 42 trials, control block with 60 trials). The Noisy Bayesian model includes noise and a rare-ball weight, ρ, that varies across subjects. The Noisy Bayesian Set ρ model (set ρ) assumes that ρ equals the ideal observer’s rare-ball weight (ρIO). The Prior Bayesian model (Prior) includes a jar bias (prior), and assumes ρ = ρIO. The Asymmetric (Asym) model assumes an asymmetric repetition bias following a low-jar response. The Prior with Variable ρ (Prior var ρ) model is the noisy Bayesian model with biased prior. The Windowing (Wind) model assumes a set window of evidence for each trial. The Variable Rare Ball (Var Rare) model sets the probability of response for the high jar based on whether or not the number of observed rare balls meets some threshold. The Rare Ball model (Rare) is a reduction of the Variable Rare Ball model and sets the rare ball threshold to 1 (observing any rare ball corresponds with a high jar response of probability Prare). The History Dependent Rare Ball (HD Rare) model incorporates past trial responses into the Rare Ball model. Under the Guess model (Guess), the high jar is chosen with some probability that is set as a free parameter, regardless of the balls observed. Models were included in subject analyses only if synthetic responses were identifiable above 80% for all blocks (Asym and Prior var ρ excluded) and if > 5 subjects were best fit by the model in any given block (Wind and HD rare models were excluded from further analyses). (TIF) [file pcbi.1010323.s012.tif]

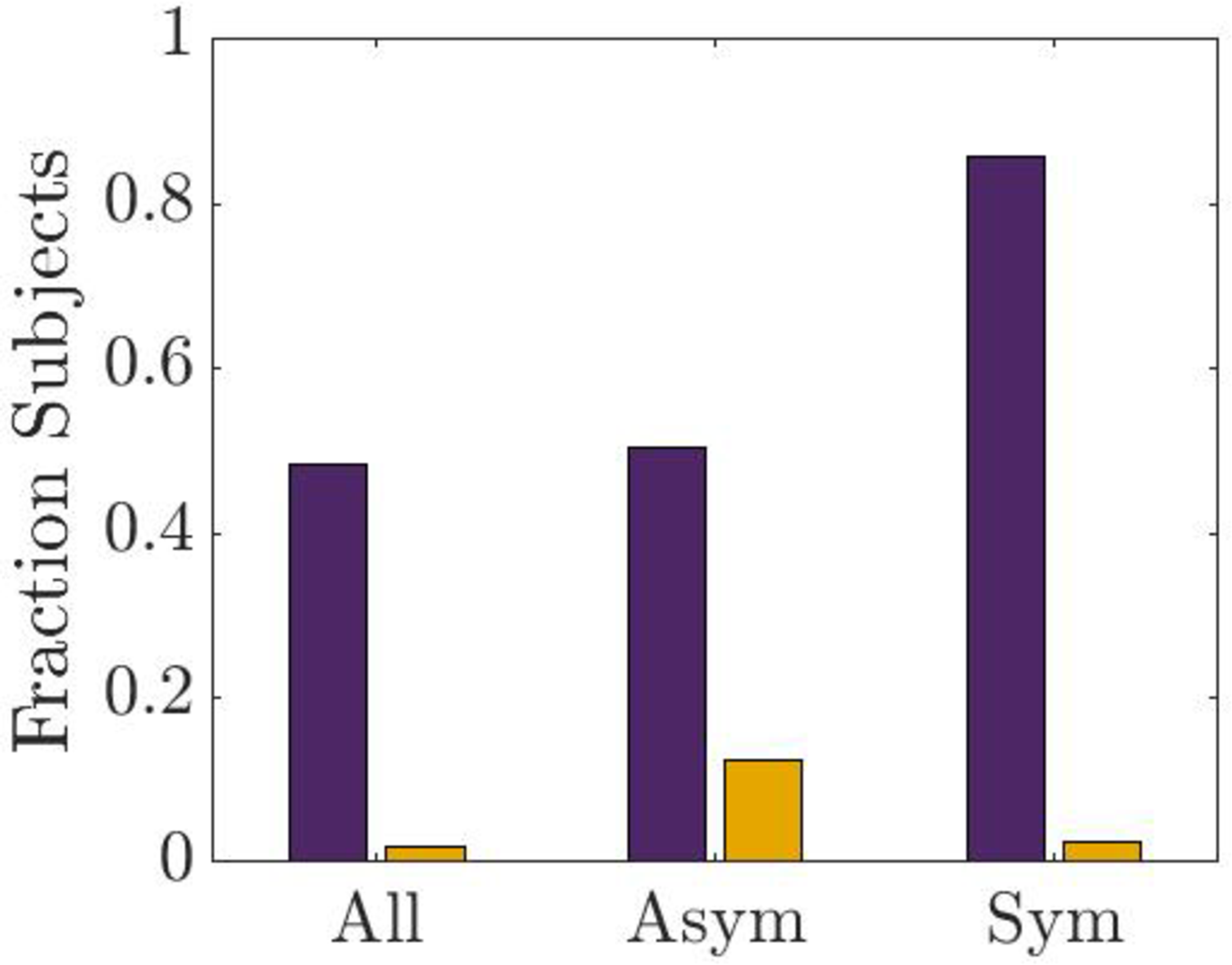

Supplement: S6 Fig — Fraction of subjects who were best fit by models in the same class, Bayesian (purple) or Heuristic (yellow). Subjects’ best-fit strategies were compared across all blocks (All), only asymmetric blocks (Asym) or only symmetric blocks (Sym). Subjects were typically best described by different models within the model class for each block. (TIF) [file pcbi.1010323.s013.tif]

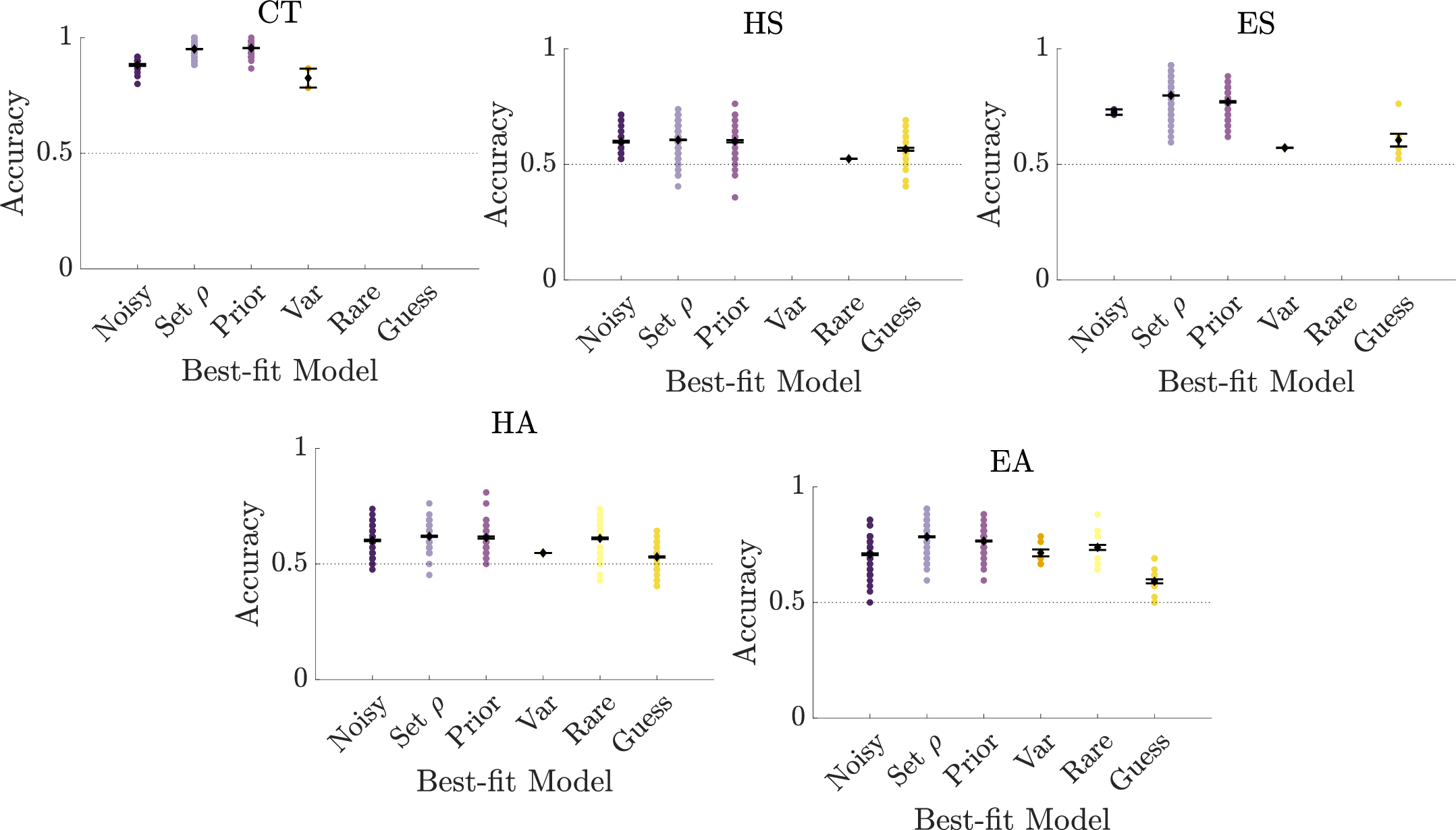

Supplement: S7 Fig — Subject accuracy based on each subject’s best-fit model in a block: Control (CT), Hard Asymmetric (HA), Hard Symmetric (HS), Easy Asymmetric (EA), Easy Symmetric (ES). Colored dots represent individual subject accuracy. Black diamonds and errorbars show the bootstrapped means (1000 iterations) and 95% confidence interval for each model-block. Accuracy was significantly (p < 0.05) above chance (0.5) for all models. (TIF) [file pcbi.1010323.s014.tif]

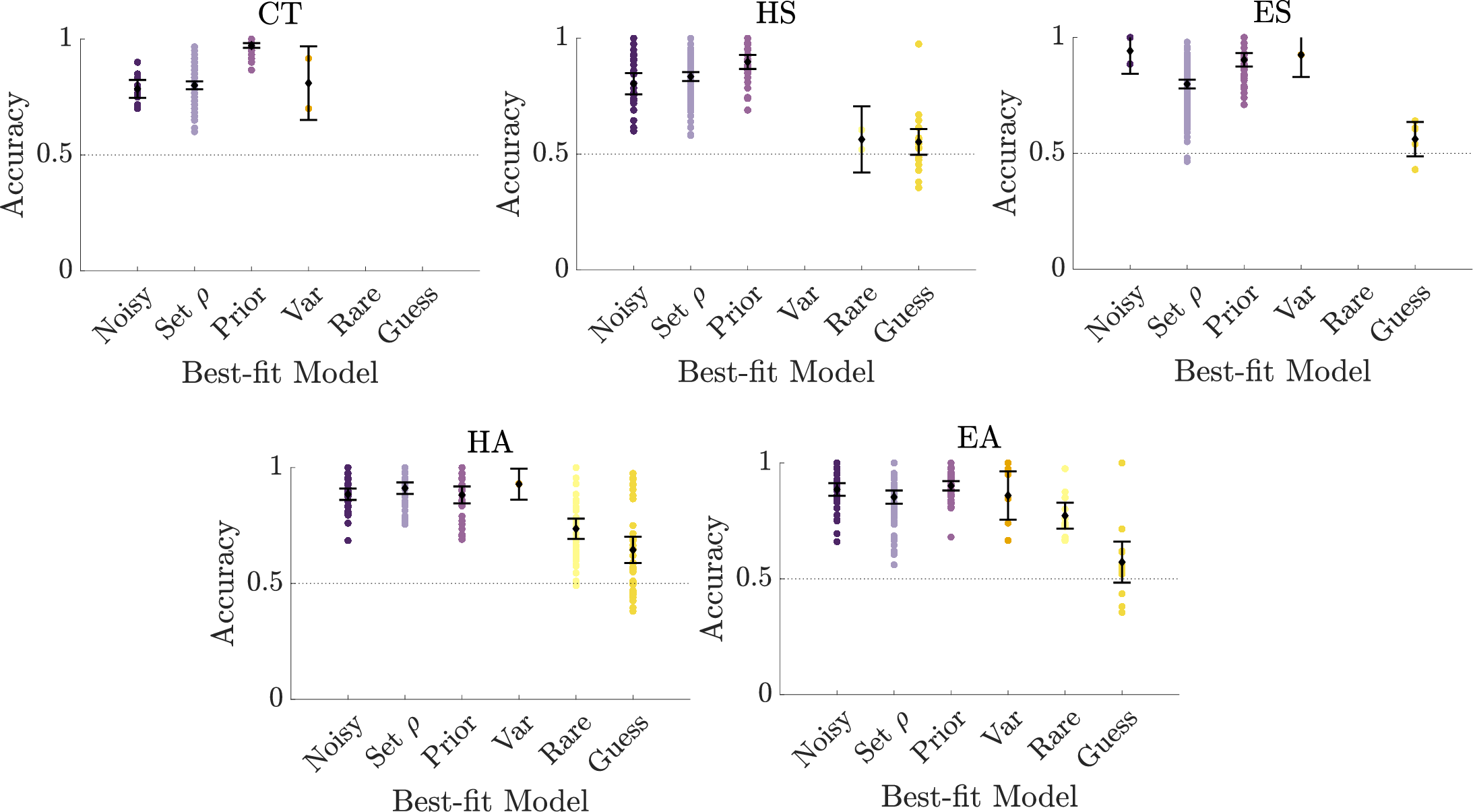

Supplement: S8 Fig — 10-fold 90/10 cross-validation accuracy performed between each subject and the model that best describes their responses for each block: Control (CT), Hard Asymmetric (HA), Hard Symmetric (HS), Easy Asymmetric (EA), Easy Symmetric (ES). Each colored point represents one individual. Black diamonds and errorbars show the bootstrapped means (1000 iterations) and 95% confidence interval for each model-block. Cross-validation accuracy was significantly above chance (0.5; p < 0.05) for all models except the Rare-Ball model in the HS block and the Guess model in all blocks. Mean cross-validation accuracy was ≥ 0.8 for all models except the Rare-Ball and Guess model. Ranges (across blocks) for the percentage of subjects with ≥ 80% cross validation accuracy for each model: Noisy Bayesian (Noisy): 40-100%; Noisy Bayesian Set ρ (set ρ): 54-85%; Prior Bayesian (Prior): 76-100%; Variable Rare Ball (Var): 50-100%; Rare Ball (Rare): 0-38%; Guess: 0-22%. (TIF) [file pcbi.1010323.s015.tif]

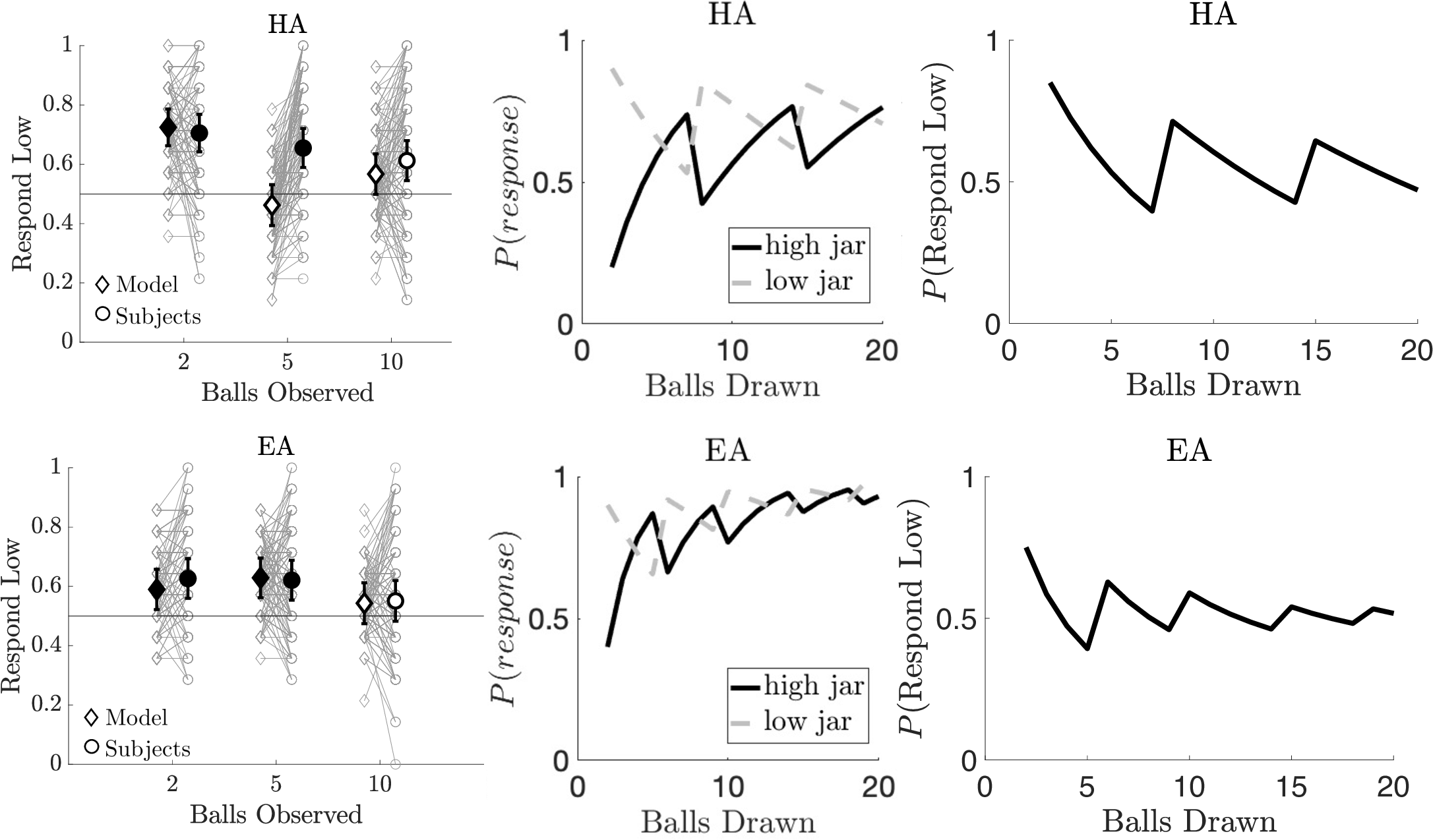

Supplement: S9 Fig — Left: Low-jar response fractions as sample lengths (number of balls observed) changes for subjects and sample-matched ideal observer (model) for asymmetric blocks (Hard Asymmetric (HA), Easy Asymmetric (EA)). Bold markers and errorbars are bootstrapped means and 95% confidence intervals. Filled markers denote a significant population shift away from 0.5 (p < 0.05). Center: For the asymmetric blocks, the ideal observer’s probability of responding correctly in favor of the low or high jar changes with the number of balls drawn and the jar asymmetries (h±). As the likelihood of observing a rare ball increases, the probability of choosing the low jar decreases, until reaching a discrete shift in the number of rare balls that must be drawn (e.g., 1 up to 2) to trigger a “high” response, generating a sawtooth-shaped response fraction function of ball number. Right: The overall (correct and incorrect trials) low-jar response probability for the ideal observer shows a general decrease in choice asymmetry as sample size increases. However, the effect is accompanied by the sawtooth structure depicted in the center panels. (TIF) [file pcbi.1010323.s016.tif]

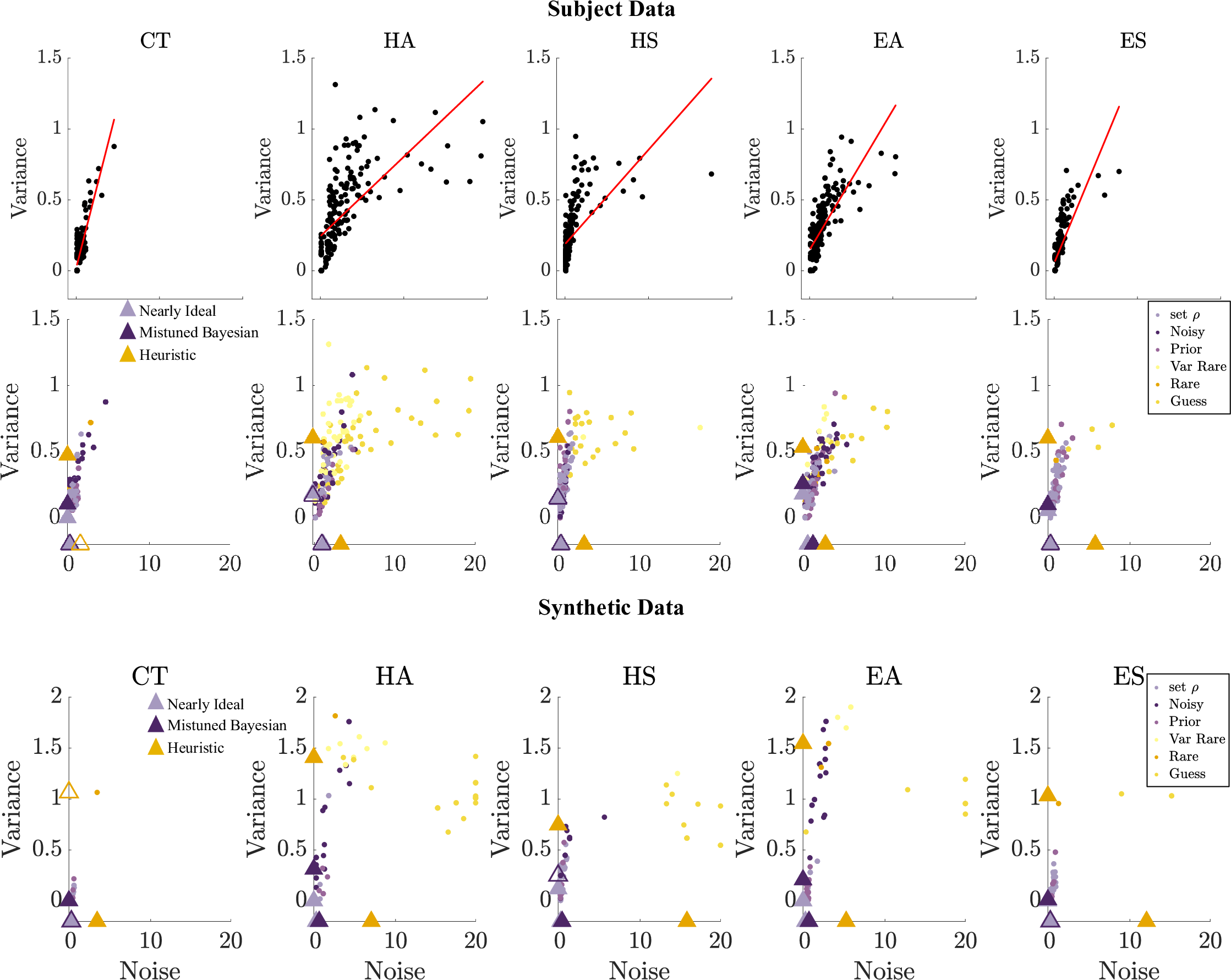

Supplement: S10 Fig — Top: Estimated noise and variance from psychometric functions fit to individual subject data (points). Noise and variance showed a significant correlation in all blocks: Control (CT), Hard Asymmetric (HA), Hard Symmetric (HS), Easy Asymmetric (EA), Easy Symmetric (ES) (Spearman’s Correlation, p < 0.05). Center: Same data as in the top row, but color coded by each subject’s best-fit models for each block. In general, heuristic subjects had the largest values of variance and noise. Triangles represent medians for each model group. Filled triangles differ significantly from the Nearly Ideal subjects (two-sided Wilcoxon rank-sum test, p < 0.05). Bottom: Noise and variance values from synthetic responses generated by each subject’s best-fit model and parameters (198 sets of synthetic responses distributed across models based on the subject strategies from Fig 4D). Both subject and synthetic data showed similar relationships between noise and variance, with Bayesian models displaying less noise and variance than heuristics. For all plots, large noise values (>20) were rescaled to 20 for visualization purposes. (TIF) [file pcbi.1010323.s017.tif]

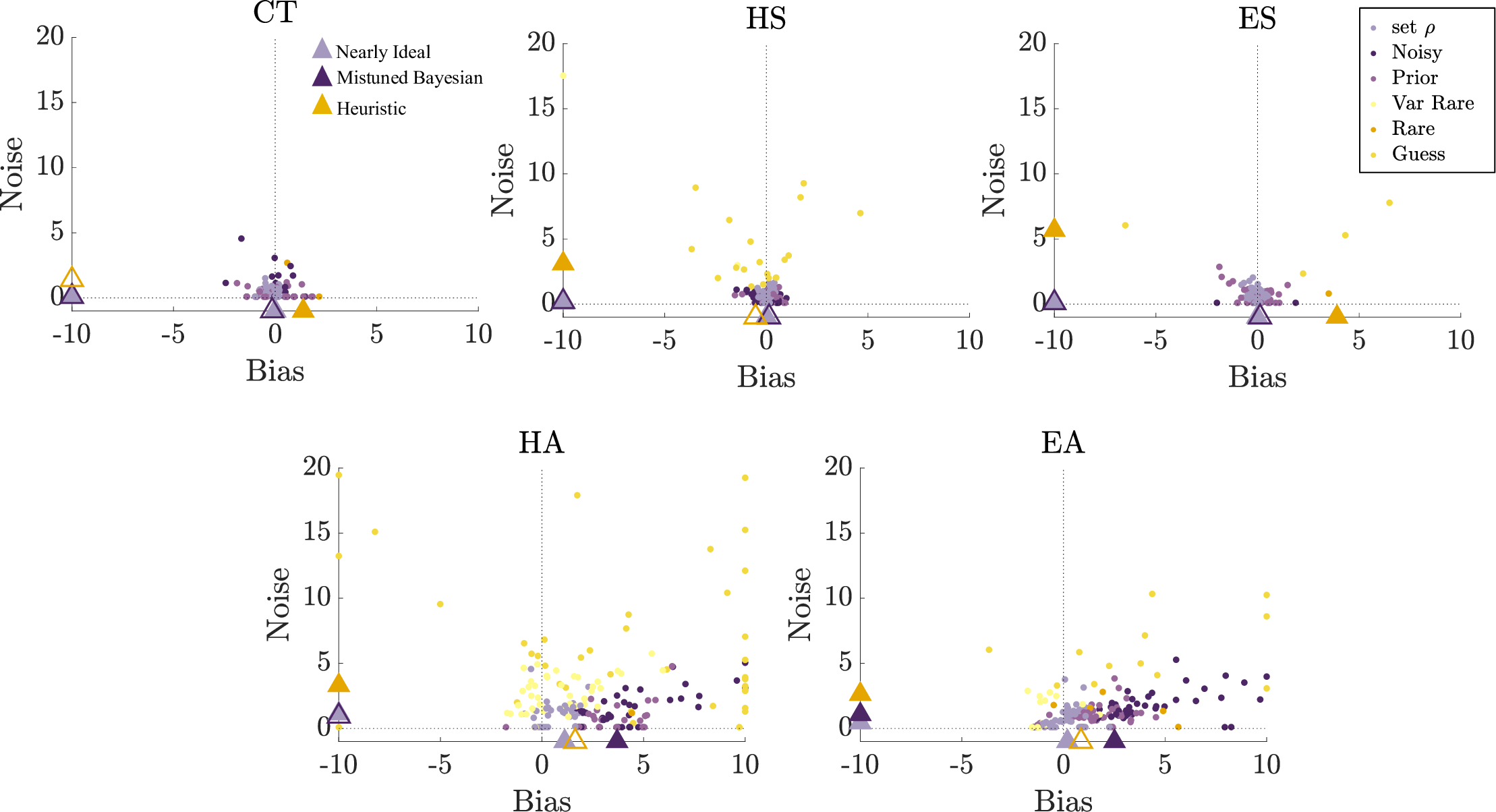

Supplement: S11 Fig — Subjects’ estimated bias and noise based on the best-fit psychometric functions shown for each task block: Control (CT), Hard Asymmetric (HA), Hard Symmetric (HS), Easy Asymmetric (EA), Easy Symmetric (ES). Here, dots represent individual subjects, color coded by an individual’s best-fit strategy. Triangles represent medians for each model group: the Nearly Ideal subjects, Mistuned Bayesian subjects, and Heuristic subjects. Filled triangles significantly differed from the Nearly Ideal subjects based on a two-sided Wilcoxon rank-sum test with p < 0.05. Large noise values (> 20) were rescaled to 20 for visualization purposes. Results mimicked those observed when using our measure of variance (see main text) instead of noise. (TIF) [file pcbi.1010323.s018.tif]

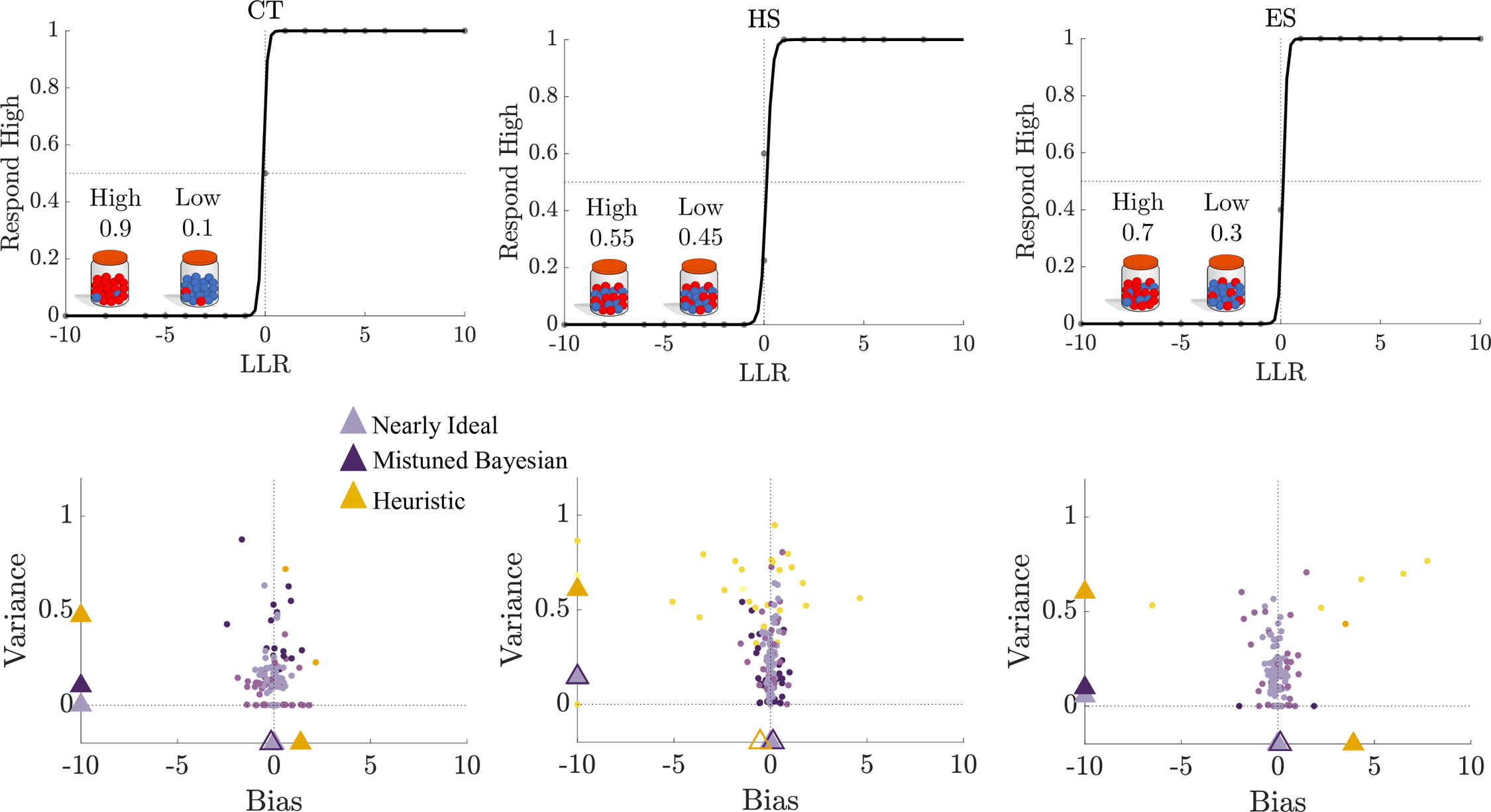

Supplement: S12 Fig — Subject bias and variance on symmetric blocks: Control (CT), Hard Symmetric (HS), and Easy Symmetric (ES), as in Figs 3C–3F and 5. Top: Median high-jar responses (points) and best-fitting logistic psychometric functions. Bottom: Bias and variance based on the best-fit psychometric function. Points reflect individual subjects, color-coded by subjects’ best-fit models. Triangles represent medians for each model group: the Nearly Ideal subjects, Mistuned Bayesian subjects, and Heuristic subjects. Filled triangles significantly differed from the Nearly Ideal subjects based on a two-sided Wilcoxon rank-sum test with p < 0.05. (TIF) [file pcbi.1010323.s019.tif]

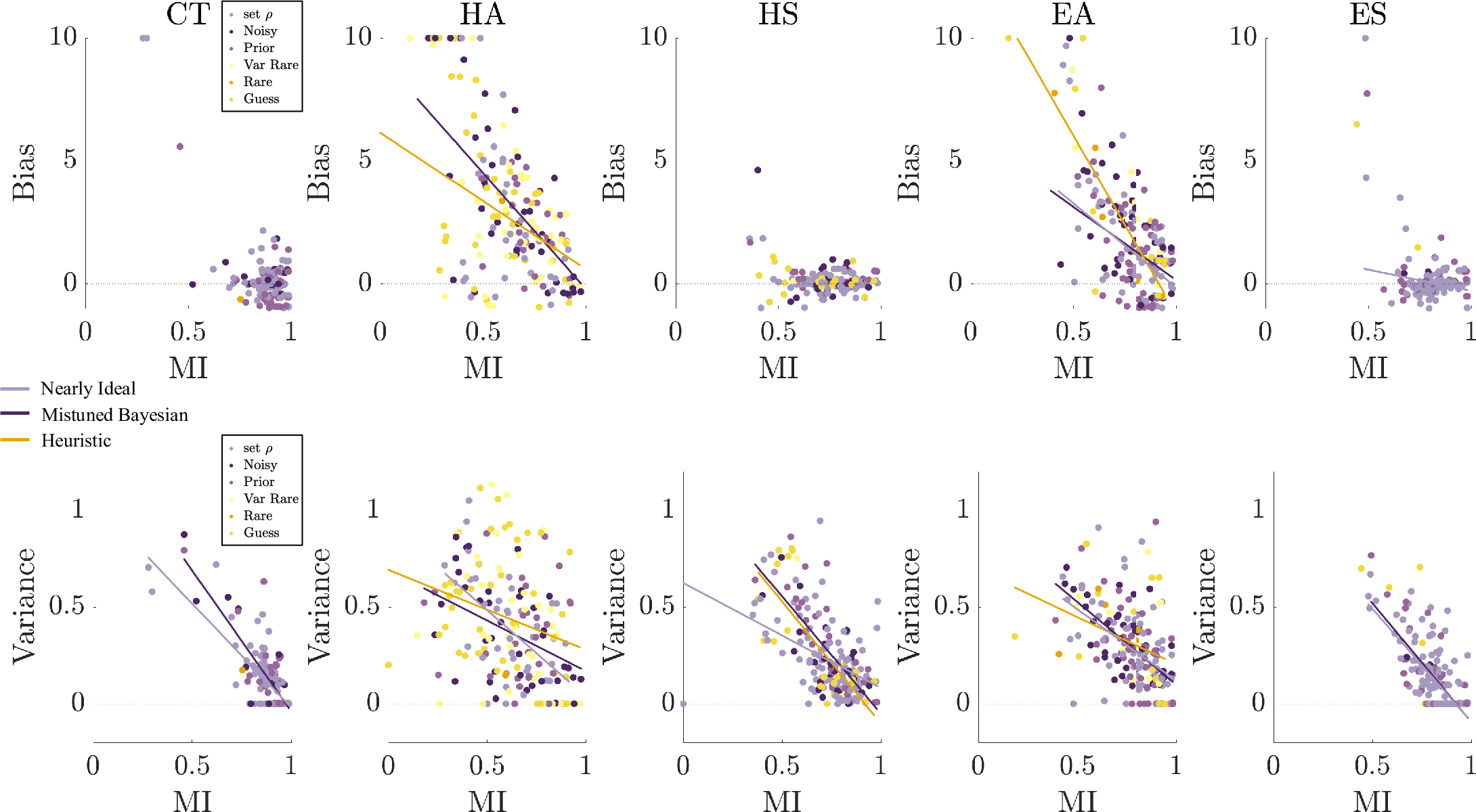

Supplement: S13 Fig — Bias and variance tended to decrease with complexity (MI) across subjects grouped by strategy, particularly on asymmetric blocks. Top: bias-MI plots as in Fig 6C for all blocks (columns, as indicated). Points are data from individual subjects, color coded by their best-fit strategy. Significant correlations (Spearman correlation, p < 0.05) are shown for each model group using color-coded lines. Only asymmetric blocks showed significant (negative) correlations, implying that within groups, bias tended to increase with decreasing strategic complexity. Bottom: variance-MI plots as in Fig 6D for all blocks, plotted as in the top row. All blocks showed at least one within-group relationship between complexity and variance, consistent with general trends of better (less variable) performance associated with more-complex strategies. (TIF) [file pcbi.1010323.s020.tif]

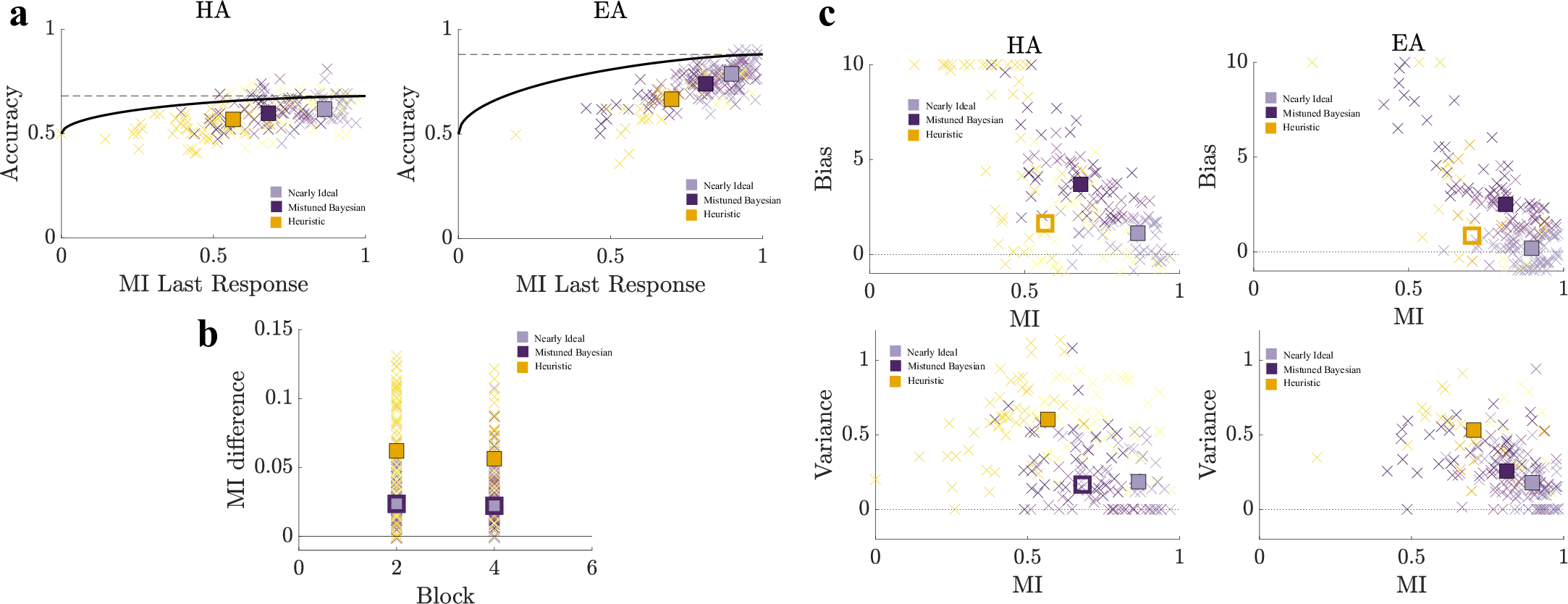

Supplement: S14 Fig — Across-group bias-variance relationships were robust to a measure of mutual information (MI) that took into account not just the balls observed on the current trial (i.e., relevant information, as in Fig 6A)) but also the previous choice (i.e., irrelevant information), for the two asymmetric blocks (columns, as indicated). a: Accuracy versus MI. The bound is the maximum accuracy attainable by the idea observer for a fixed MI in the limit of many trials. Note that points could exceed the asymptotic accuracy bound because the number of trials for each subject was finite. The dashed horizontal lines indicate the accuracy bound for maximum MI values. X’s are data from individual subjects. Squares are per-group medians (filled symbols for Mistuned Bayesian and Heuristic groups indicate that the median MI is significantly different from that of the Nearly Ideal group median, Wilcoxon rank-sum test, p < 0.05). Including past choices tended to give slightly higher MI measures but maintain the same ordering from Heuristics (simplest), to Mistuned Bayesian, to Nearly Ideal (most complex; compare to Fig 6A)).b: Difference in MI using this measure versus MI without the previous choice. X’s are data from individual subjects. Squares are per-group medians (filled symbols for Mistuned Bayesian and Heuristic groups indicate that the ordinate value is significantly different from that of the Nearly Ideal group median, Wilcoxon rank-sum test, p < 0.05). In general, including the previous choice increased MI (i.e., subjects tended to have sequential choice dependencies) but did not affect the inverted bias-variance trade-off.c: Bias-MI and variance-MI plots using this MI measure that includes the previous choice. (TIF) [file pcbi.1010323.s021.tif]

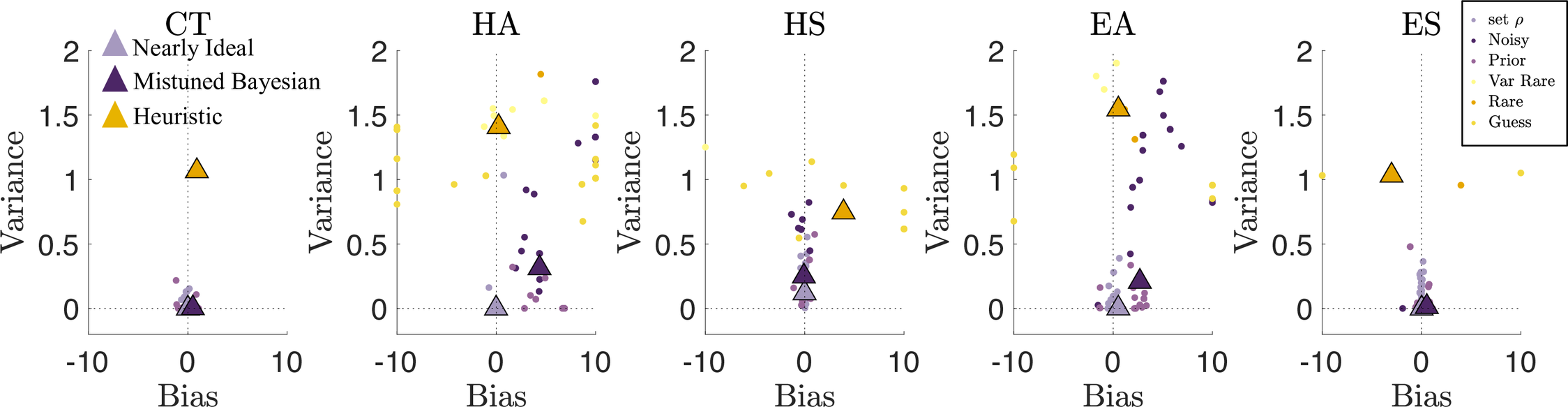

Supplement: S15 Fig — Synthetic sets of responses were produced using each subject’s best-fit model and parameters and new samples of ball draws (198 sets of synthetic responses distributed across models based on the strategies that best describe subjects’ responses from Fig 4D) for each block: Control (CT), Hard Asymmetric (HA), Hard Symmetric (HS), Easy Asymmetric (EA), Easy Symmetric (ES). Synthetic responses were then fit to psychometric functions with bias and variance values extracted. Each dataset of synthetic responses is denoted by a colored point associated with the generating model. Triangles show medians for each group. In asymmetric blocks, Mistuned Bayesian models show bias. (TIF) [file pcbi.1010323.s022.tif]

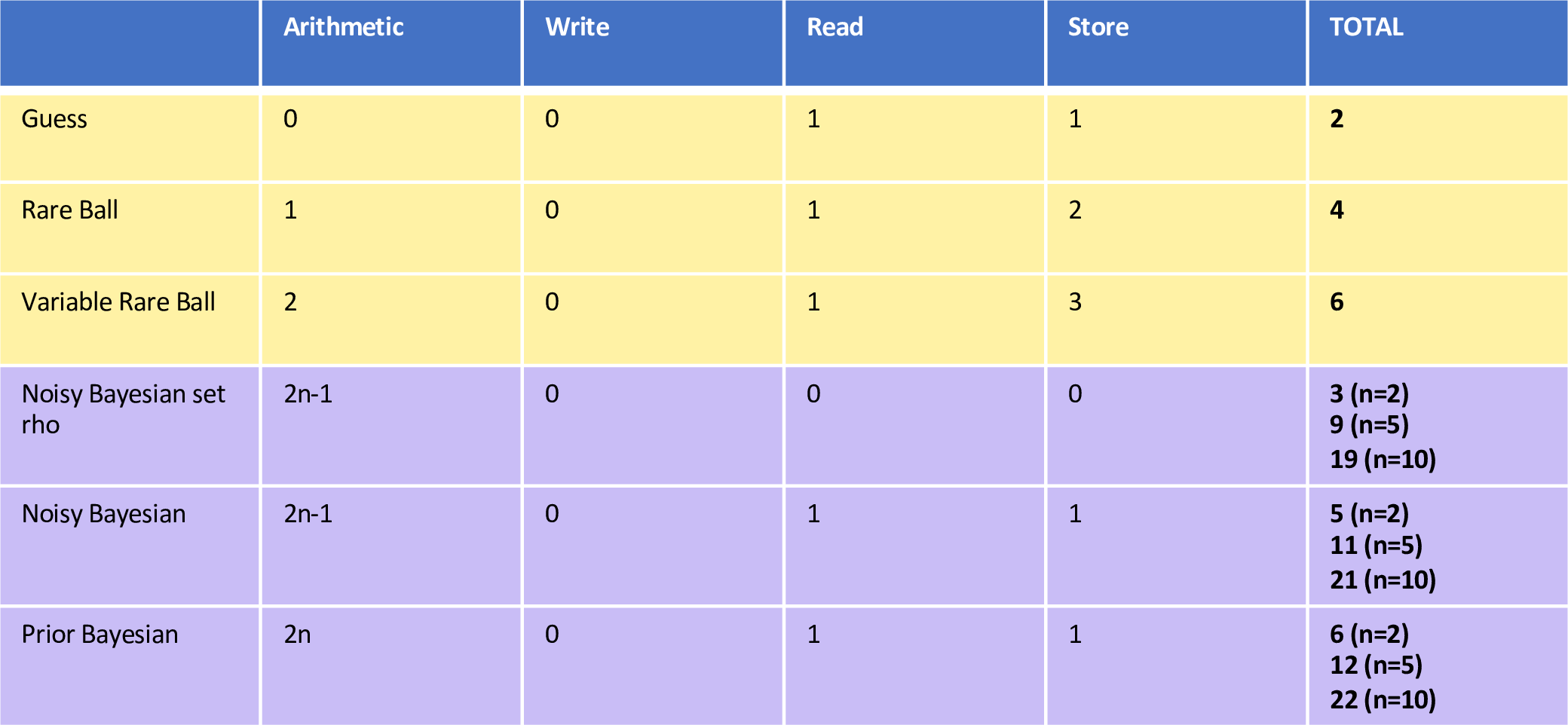

Supplement: S16 Fig — Algorithmic complexity [9] for each model was computed based on the number of operations performed on a trial, broken into: arithmetic, writing to memory, reading from memory, and storage operations. Heuristic models have lower complexity (yellow) compared to Bayesian models (purple). Bayesian model complexity varies with the number of balls observed (n). Example computations are shown for sample lengths of 2,5, and 10 balls. Computations were based on the following operations involved in each strategy: Guess: Read and store parameter Pguess.Rare Ball: Identify presence of the rare ball (max), read the probability of response, store Prare and Pno.Variable Rare Ball: All elements from the Rare-Ball model with additional operations to compute the number of rare balls and store the rare-ball threshold θ.Noisy Bayesian Set ρ: Multiplication of the ball weight for each ball observed (n) and n − 1 summations.Noisy Bayesian: Arithmetic as in the Noisy Bayesian Set ρ model with additional operations to read and store the rare-ball weight ρ.Prior Bayesian Arithmetic as in the Noisy Bayesian model with inclusion of the prior that is read and stored. (TIF) [file pcbi.1010323.s023.tif]
